# Unraveling the Genotypic and Phenotypic Diversity of the Psychrophilic *Clostridium estertheticum* Complex, a Meat Spoilage Agent

**DOI:** 10.3389/fmicb.2022.856810

**Published:** 2022-03-28

**Authors:** Joseph Wambui, Marc J. A. Stevens, Nicole Cernela, Roger Stephan

**Affiliations:** Institute for Food Safety and Hygiene, Vetsuisse Faculty, University of Zurich, Zurich, Switzerland

**Keywords:** *Clostridium estertheticum*, *Clostridium tagluense*, comparative genomics, meat spoilage, hydrogenase, flagella, carbohydrates

## Abstract

The spoilage of vacuum-packed meat by *Clostridium estertheticum* complex (CEC), which is accompanied by or without production of copious amounts of gas, has been linked to the acetone–butyrate–ethanol fermentation, but the mechanism behind the variable gas production has not been fully elucidated. The reconstruction and comparison of intra- and interspecies metabolic pathways linked to meat spoilage at the genomic level can unravel the genetic basis for the variable phenotype. However, this is hindered by unavailability of CEC genomes, which in addition, has hampered the determination of genetic diversity and its drivers within CEC. Therefore, the current study aimed at determining the diversity of CEC through comprehensive comparative genomics. Fifty CEC genomes from 11 CEC species were compared. Recombination and gene gain/loss events were identified as important sources of natural variation within CEC, with the latter being pronounced in genomospecies2 that has lost genes related to flagellar assembly and signaling. Pan-genome analysis revealed variations in carbohydrate metabolic and hydrogenases genes within the complex. Variable inter- and intraspecies gas production in meat by *C. estertheticum* and *Clostridium tagluense* were associated with the distribution of the [NiFe]-hydrogenase *hyp* gene cluster whose absence or presence was associated with occurrence or lack of pack distention, respectively. Through comparative genomics, we have shown CEC species exhibit high genetic diversity that can be partly attributed to recombination and gene gain/loss events. We have also shown genetic basis for variable gas production in meat can be attributed to the presence/absence of the *hyp* gene cluster.

## Introduction

*Clostridium estertheticum* complex (CEC) comprises 10 closely related psychrophilic and anaerobic species (Palevich et al., 2021; Wambui et al., 2021a). Members of CEC have been isolated from different ecological niches including terrestrial and aquatic niches but, the majority of the species have been isolated from meat or meat processing facilities due to their involvement in meat spoilage (Collins, 1992; Spring, 2003; Suetin et al., 2009). In particular, *C. estertheticum* is the main causative agent of blown pack spoilage (BPS) of meat, which is characterized by pack distention arising from production of copious amounts of hydrogen and carbon dioxide (Wambui and Stephan, 2019). On the other hand, other species, such as *Clostridium tagluense,* have been linked with meat spoilage accompanied by little or no gas production (Dorn-In et al., 2022).

Microbial meat spoilage is usually attributed to an abundance of utilizable substrates including glycogen, lactate, amino acids, and low levels of glucose (Nychas et al., 2008). The model CEC species causing BPS, *C. estertheticum*, first utilizes glucose in the meat and once this substrate is exhausted, it switches to lactate utilization, which results in production of H_2_ and CO_2_ (Yang et al., 2009). The absence of ammonia and sulfur dioxide production by *C. estertheticum* suggests carbohydrates and lactate in meat are preferentially utilized over amino acids (Yang et al., 2009, 2010). Therefore, BPS has been directly linked to the utilization of fermentable substrates in meat. Compared to *C. estertheticum*, data on substrate utilization by other CEC species are limited, which further limits our understanding on the collective contribution of CEC to meat spoilage. The phenotypic data that are available for *C. tagluense*, suggest the species has a more narrow substrate spectrum than *C. estertheticum* (Suetin et al., 2009). The illustrated phenotypic heterogeneity among CEC species is indicative of genotypic diversity that may have an influence on metabolic pathways that are linked to meat spoilage.

The utilization of substrates coupled with the production of H_2_ and CO_2_ and other metabolites, such as acetate, butyrate, formate, butanol, and ethanol (Yang and Badoni, 2013) suggests that CEC species are capable of acetone–butyrate–ethanol fermentation, which can be linked to meat spoilage. Similarly, acetone–butyrate–ethanol fermentation has been linked with late blowing defect in hard and semi-hard cheese as a result of metabolism of lactate and other substrates by *Clostridium tyrobutyricum*, *Clostridium butyricum* and other clostridia resulting in the production of H_2_, CO_2_, and organic acids, mainly butyric acid, accompanied by abnormal aroma and flavor (Bassi et al., 2015; Gómez-Torres et al., 2015). The acetone–butyrate–ethanol fermentation is a biphasic process involving acidogenic and solventogenic phases. In the first phase, an organism converts diverse substrates in a typical acidogenic fermentation process consequently producing butyrate, acetate, H_2_, and CO_2_ (Patakova et al., 2013). The solventogenic phase occurs as the organism transitions to stationary phase, and it is characterized by growth cessation, a switch to butanol, acetone, and ethanol production and the initiation of endospore formation (Khamaiseh et al., 2014). The metabolic switch is necessary to avoid the deleterious effects of acid accumulation and to allow sufficient time for endospore formation (Zheng et al., 2009). Therefore, acetone–butyrate–ethanol fermentation is a critical and complex metabolic process needed for the development of acidogenic and solventogenic clostridia, such as CEC.

Comparative genomics allows multiple bacterial genomes to be more efficiently and conveniently compared in order to reveal inter- and intraspecies similarities or differences in particular groups of interest. Furthermore, it allows for better functional analysis at the genetic level (Yu et al., 2018). In this regard, a previous comparative genomic study involving six strains from four CEC species, *C. estertheticum*, *C. tagluense*, genomospecies1, and genomospecies2, identified genes involved in acetone–butyrate–ethanol fermentation and provided the first genomic link to the fermentation-mediated meat spoilage by CEC (Palevich et al., 2021). Although the fermentation has been linked with meat spoilage, the exact mechanism that results in variable gas production in chilled vacuum-packed meat has not been elucidated. Recently, more CEC genomes have been published (Wambui et al., 2020b, 2021a, 2022) thus providing an opportunity for comprehensive comparative genomics through which the genetic diversities within CEC can be assessed to reveal the underlaying causes of variable meat spoilage at the genomic level. At the same time, other unknown inter- and intraspecies genotypes of CEC can be unraveled. As a prerequisite to the analyses, it is imperative that efforts are made to increase the number of sequenced CEC genomes. Therefore, the current study aimed at (i) detecting, isolating, and sequencing new CEC strains from the meat processing environment and (ii) determining the genetic diversity of CEC through comprehensive comparative genomics.

## Materials and Methods

### qPCR-Based Screening of *Clostridium estertheticum* Complex Strains

Four different sample types which included meat processing equipment (*n* = 90), bovine fecal samples (*n* = 3), and meat juice samples of unspoiled meat (*n* = 153) or BPS meat (*n* = 3) were screened for the presence of CEC by quantitative real-time PCR (qPCR) as previously described (Wambui et al., 2020c). Briefly, sponge samples obtained from the meat processing equipment were homogenized with Reinforced Clostridia Media (RCM; 25 ml) for 60 s using a stomacher. Hungate tubes containing 10 ml of the homogenized meat processing equipment sample were heated at 80°C for 10 min. The meat processing equipment and meat juice samples of unspoiled meat were incubated anaerobically for 10–14 days at 8°C prior to screening while meat juice samples from BPS samples were screened immediately after sampling. Current and subsequent anaerobic incubations were carried out in rectangular anerobic boxes (7.0 L; bioMérieux, Inc., Marcy l’Etoile, France), and the anaerobic conditions were generated by three 2.5 L AnaeroGen Sachets (Thermo Fisher Scientific, Waltham, MA, United States) per box. DNA was extracted from 100 μl of each sample using the MagNa Pure LC DNA Isolation Kit III (Roche, Rotkreuz, Switzerland) by the MagNa Pure LC robotic workstation (Roche). Each sample was mixed with 10 μl lysozyme (20 mg/ml in phosphate-buffered saline) solution and incubated at 37°C for 30 min. Subsequent steps from lysis with proteinase K to DNA elution were carried out according to the instruction manual of the DNA extraction kit. The qPCR primers, probes and protocol used herein were as previously described (Brightwell and Clemens, 2012). qPCR-positive meat processing equipment and meat juice samples of unspoiled meat were enriched anaerobically 4 weeks at 8°C. The bovine fecal samples were obtained from a previous study (Wambui et al., 2021a) where attempts to isolate CEC strains were unsuccessful and thus had been stored anaerobically at 4°C.

### Isolation and Presumptive Identification of CEC Strains

Isolation of CEC from meat juice samples of BPS samples was carried out by direct plating. Briefly, the samples were serially diluted 10-fold in saline solution and plated on Columbia agar supplemented with 5% defibrinated sheep blood (CBA). Isolation of CEC from the enriched meat processing equipment, bovine fecal samples, and meat juice samples of unspoiled meat was carried out after elimination of competitive microflora and activation of CEC spores as previously described without modification (Wambui et al., 2021b). Colonies on CBA displaying clostridial characteristics described previously (Húngaro et al., 2016) were selected and purified anaerobically on CBA for 3 weeks at 8°C. The isolates were confirmed as members of CEC by qPCR as described above.

### DNA Extraction, Whole-Genome Sequencing, and Identification of CEC Strains

The extraction and whole-genome sequencing of CEC isolates was carried out as previously described (Wambui et al., 2020a). Briefly, the genomic DNA was isolated using the DNA blood and tissue kit (Qiagen, Hombrechtikon, Switzerland). The sequencing outputs, which were 150–300 bp pair-ended reads, were prepared using Nextera DNA Flex chemistry using the Nextera DNA Flex Sample Preparation Kit (Ill) as per manufacturer’s guidelines (Illumina, San Diego, CA, United States). The resulting transposon-based libraries were sequenced on a MiniSeq sequencer (Illumina) with a minimal coverage of 50-fold. The MiniSeq MidOutput Reagent Cartridge (300 cycles) was used. Demultiplexing and adapter trimming was done using the Miniseq local run manager version 2.4.1 using standard settings. The reads were checked for quality using FastQC (Andrews, 2010) then assembled with SPAdes v. 3.12.0 (Bankevich et al., 2012) using Shovill 1.0.9.^1^ Since *Clostridium algoriphilum* DSM 16153^T^ has not been sequenced yet, we purchased the strain from the Leibniz Institute DSMZ-German Collection of Microorganisms and Cell Cultures, revived it as previously described, and sequenced it as described above. The quality of the genomes was checked using ContEST16S (Lee et al., 2017) and CheckM (Parks et al., 2015). The complete *rpoB* gene sequences were identified using blastn and extracted from the genomes with samtools using the faidx option and the blast results as input (Altschul et al., 1990; Li et al., 2009). These and the *rpoB* sequences of 34 publicly available CEC genomes (Wambui et al., 2021a), were aligned in CLC Workbench Genomics v. 8.1 (Qiagen, Aarhus, Denmark) using default settings while the phylogenetic tree was created with the same software using the Maximum likelihood Phylogeny method whereby the neighborhood joining method using the Jukes Cantor model were applied, respectively. Bootstraps were based on 1,000 replicates. The correct species assignment of the CEC isolates was carried out *in silico* from the pairwise nucleotide comparison of Average Nucleotide Identity (ANI), which was determined with pyANI (Pritchard et al., 2016) using the BLAST algorithm. The *rpoB*-based phylogeny consisting of the Type/Representative strains of CEC, *Clostridium algidicarnis*, *Clostridium frigidicarnis*, *Clostridium gasigenes*, *Clostridium putrefaciens*, and *Clostridium argentinense* was used to validate a divergent isolate as a member of CEC. Where necessary, resulting newick files were visualized in iTOL (Letunic and Bork, 2019). Core genome MLST was performed in Seqsphere+8.2.0 (Ridom, Münster, Germany). The *C._estertheticum*_cgMLST scheme was constructed using the type strain DSM 14864^T^ as seed and all other available *C._estertheticum_*genomes as testers. Standard settings were applied for target selection.

### Characterization of the General Features of CEC Genomes and Pan-Genome Analysis

The 16 and 34 CEC genomes from the current and previous studies, respectively (Supplementary Tables 1A,B) were annotated using Prokka (Seemann, 2014) and RAST (Brettin et al., 2015). The GFF3 output files from Prokka were used for pan-genome analysis of the 50 CEC genomes in Roary v3.11.2 (Page et al., 2015) with the minimum percentage identity for BLASTP set at 60%, 70%, 80%, 90%, and 95%. For each setting, four different gene classes grouped into “core” (99% ≤ strains ≤100%), “soft-core” (95% ≤ strains ≤100%), “shell” (15% ≤ strains <95%), and “cloud” (0% ≤ strains <15%) were obtained with the -s setting for paralogues clustering. Pan-genome analysis were further carried out for the 25 *C. estertheticum* genomes in Roary with the minimum percentage identity for BLASTP set at 80%. Recombination events in CEC were estimated using Gubbins version 2.4.1. (settings: -f 30) using coreSNP alignment and tree as input (Croucher et al., 2015). For this purpose, the core genome alignment was created using Parsnp v1.5.3 (Treangen et al., 2014). The maximum likelihood phylogeny of *C. estertheticum* was determined using RAxML (Randomized Axelerated Maximum Likelihood; Stamatakis, 2014). The phylogenetic trees were visualized and annotated with metadata in iTOL (Letunic and Bork, 2019). Pan-genome characteristics were calculated using the package micropan in R using 500 the random permutations of genome ordering (Snipen and Liland, 2015).

### Comparative Genomics and Phenotypic Characterization CEC

Function annotation and classification of proteins were performed by sequence comparison against the eggNOG database 5.0 (Huerta-Cepas et al., 2019), using the eggNOG-Mapper v2 (Cantalapiedra et al., 2021). Putative enzymes involved in the breakdown, biosynthesis, or modification of carbohydrates were identified using CAZy database using dbCAN using default settings (Drula et al., 2022). The genes involved in the acetone–butyrate–ethanol fermentation were identified from the previous work on CEC (Palevich et al., 2021) and other solventogenic clostridia (Li et al., 2020). In addition, the genes annotated as hydrogenases were manually identified in the RAST annotated CEC genomes. In total, a comprehensive list of 48 genes (Supplementary Table 2) was established and each gene was manually identified and counted in each of the RAST annotated CEC genome. Where applicable, the genes and pathways were validated using the KEGG (Kyoto Encyclopedia of Genes and Genomes) database (Kanehisa et al., 2016).

For phenotypic characterization, respective cultures were initially grown anaerobically at 8°C on CBA then standardized to McFarrand 3.0. Utilization of fermentable substrates by *C. estertheticum* and *C. tagluense* was determined using the standardized cultures as previously described (Wambui et al., 2020a) using the API20A kit (bioMeriéux, Marcy l’Etoile, France) and following manufacturer’s specifications. The API20A strips were incubated at 8°C anaerobically for 2 weeks. For motility test and gas production in meat, standardized cultures were first inoculated 1:100 into RCM broth and incubated at 8°C for 10 days. The motility test was performed in liquid media which essentially determines swimming motility, requires shorter incubation time than growth on agar, and is easily reproducible. For this test, 200 μl of each culture was slowly injected into Hungate tubes containing fresh RCM broth using a syringe and the tubes incubated for 2 weeks at 8°C without shaking. Motility was determined as the movement of the cultures from the surface toward the bottom of the tubes. Gas production in meat was carried out as previously described with slight modifications (Dorn-In et al., 2022). Five hundred microliter of each culture was spread on the surface of approximately 500 g of beef sample. The samples were vacuum-packed and stored for 28 or 56 days at 8°C. Uninoculated vacuum-packed beef samples were included as negative controls. At the end of the storage period, meat spoilage was scored from 0 to 5 according to a previous description (Boerema et al., 2007). Loss of flagellar genes has been shown to increase exopolysaccharide synthesis (Watnick et al., 2001). Therefore, CEC colony morphology that could be related loss of flagellar genes, including sliminess, were determined on cultures grown on CBA for 8 weeks at 8°C. Each experiment was carried out in three biological replicates apart from the gas production in meat with two biological replicates.

### Data Analysis

Data for substrate utilization, motility test, and gas production were described qualitatively. The distribution of carbohydrate-active enzymes was described as means and SD and the means compared using the Mann–Whitney U (*p* = 0.05) with Bonferroni correction. Data analysis and visualization were carried in R Studio Version 1.1.463 (RStudio, Inc., Boston, United States).

## Results

### Identification of New Sequenced Strains, Including a Novel Species, Highlights the Diversity of CEC Species

**Figure 1:**
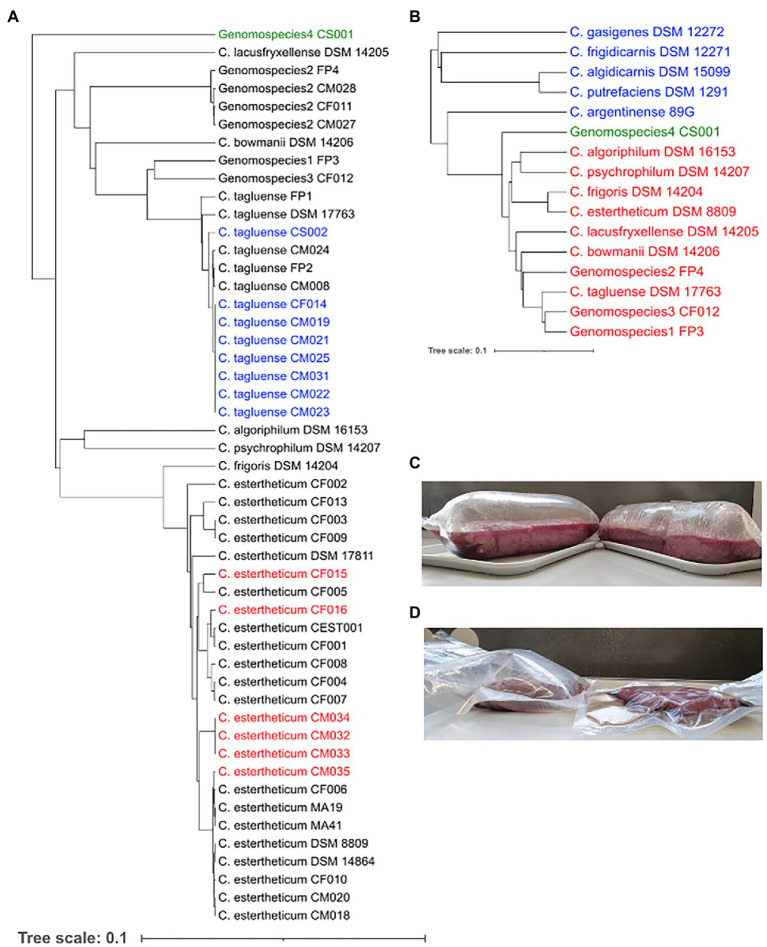
Identification of new *Clostridium estertheticum* complex (CEC) isolates. **(A)** *rpoB* gene-based phylogeny of CEC strains. Newly isolated *C. estertheticum*, *C. tagluense,* and genomospecies4 strains are highlighted in red, blue, and green text, respectively. **(B)** Genomospecies4 CS001 (green) was confirmed as a novel member of CEC using the *rpoB* gene-based phylogenetic analysis of type/representative strains of CEC (red) and other related *Clostridia* spp. (blue). **(C)** Blown pack spoilage cases of horse meat that were likely caused by *C. estertheticum* CM033 and CM034. **(D)** Blowing ability of *C. estertheticum* CM034 (left) in beef against uninoculated beef (right).

In an effort to expand the existing collection of sequenced CEC strains, 15 new strains, which were confirmed by qPCR, were isolated from meat processing equipment (*n* = 2), bovine fecal samples (*n* = 3), and meat juice samples of unspoiled meat (*n* = 7) and BPS meat (*n* = 3). Based on the *rpoB* gene phylogeny (Figure 1A), 14 strains were identified as *C. tagluense* (*n* = 8) and *C. estertheticum* (*n* = 6). Their correct species identification was confirmed and validated by ANIb analysis (Supplementary Figure 1). The ANIb between the *C. tagluense* strains and *C. tagluense* DSM 17763^T^ ranged between 97.27% and 97.32% while the ANIb values between the *C. estertheticum* strains and *C. estertheticum* DSM 8809^T^ ranged from 95.80% to 98.18% confirming the correct species identification of the strains. Strain, CS001, formed an outgroup compared to other known 49 CEC strains (Figure 1A) suggesting a novel species within the CEC. Interspecies ANIb between CS001 and the other CEC strains ranged from 78.60% to 81.36% for *C. psychrophilum* DSM 14207^T^ and *C. tagluense* DSM 17763^T^, respectively. Furthermore, strain CS001 had an ANIb of <81.7% with any of the 122 representative genomes of the genus *Clostridium* in the NBCI database, confirming it is a novel CEC species. The new species is herein referred to as genomospecies4. *rpoB*-based phylogenetic reconstruction with 16 type/representative strains from 11 CEC species, four non-CEC *Clostridium* species that are commonly isolated from meat (*C. algidicarnis*, *C. frigidicarnis*, *C. gasigenes*, and *C. putrefaciens*) and *C. argentinense*, which is the known closest *Clostridium* species to CEC, further provided evidence that genomospecies4 is a novel species within CEC despite its high divergence (Figure 1B). The novel species shows CEC is both expansive and diverse.

The strains, CM032, CM033, and CM034, isolated from BPS cases (Figure 1C; Supplementary Figure 2) were identified as *C. estertheticum*, and clustered together (Figure 1A). cgMLST of species *C. estertheticum* demonstrated the three strains had SNP differences of two to three, indicating that they are clonal (Supplementary Figure 3). Interestingly, strains CM033 and CM034 were isolated from BPS involving horse meat (Figure 1C), while their involvement in BPS was validated with CM034, which caused BPS in vacuum-packed beef (Figure 1D; Table 1). To the best of our knowledge, this is the first reported case of BPS in horse meat caused by *C. estertheticum*. Phenotypic analysis revealed CS001 from the novel species caused meat spoilage with a score of 3 which corresponds with blown puffy packs (Table 1). We further compared the new species with the other recently described species, genomospecies2 and genomspecies3 whose representative strains, CM028 and CF012, caused spoilage with a score of 3 and 2, respectively with the latter score corresponding to loss of vacuum without evidence of substantial gas production. This further demonstrates the significant involvement in meat spoilage of not only the known CEC species, but also other newly described species.

**Table 1:** Blown pack spoilage (BPS) potential of selected *Clostridium estertheticum* complex (CEC) isolates.

Strain	Species	BPS score^†^	Characteristic
CM034	*C. estertheticum*	4	Fully distended pack, but not tightly stretched
CM028	Genomospecies2	3	Blown puffy pack
CF012	Genomospecies3	2	Loss of vacuum within the pack
CS001	Genomospecies4	3	Blown puffy pack
DSM 14864^*^	*C. estertheticum*	5	Tightly stretched overblown pack
Negative control (no inoculum)		0	No gas bubbles in the drip

* Positive control.

† Mean of two biological replicates.

*Clostridium algoriphilum* DSM 16153^T^ was also sequenced within this study. On the *rpoB* phylogeny (Figure 1A), the strain was closely related to *C. psychrophilum* DSM 14207^T^. This was further confirmed by the ANIb of 84.63% between the two genomes. *Clostridium algoriphilum* DSM 16153^T^ was distantly related to the newly identified genomospecies4 CS001 (ANIb = 79.53%).

### Genome and Pan-Genome Analyses Reveal High Genetic Diversity Within CEC

Although CEC forms a single clade within the *Clostridium* genus, their genomes demonstrated high variability with respect to size, number of coding sequences (CDS), and G + C content (Supplementary Table 1). The size varied from 3.662 Mbp in genomospecies2 CM028 to 5.555 Mbp in genomospecies1 FP3 while the CDS ranged from 3.337 in genomosspecies2 CM028 to 5.271 in *C. tagluense* FP2. The G + C content ranged from 29.9% in *C. psychrophilum* DSM 14207 to 31.7% in genomospecies2 CM027 and genomospecies3 CF012. The size, CDS and G + C of CS001 from the newly identified species, genomospecies4, were 4.139 Mbp, 3,831 and 31.6%, respectively, and 4.520 Mbp, 4,524, and 30.7%, respectively, in the newly sequenced *C. algoriphilum* DSM 16153^T^. Overall, genomospecies2 has the smallest genome size within CEC (Supplementary Figure 4).

**Figure 2:**
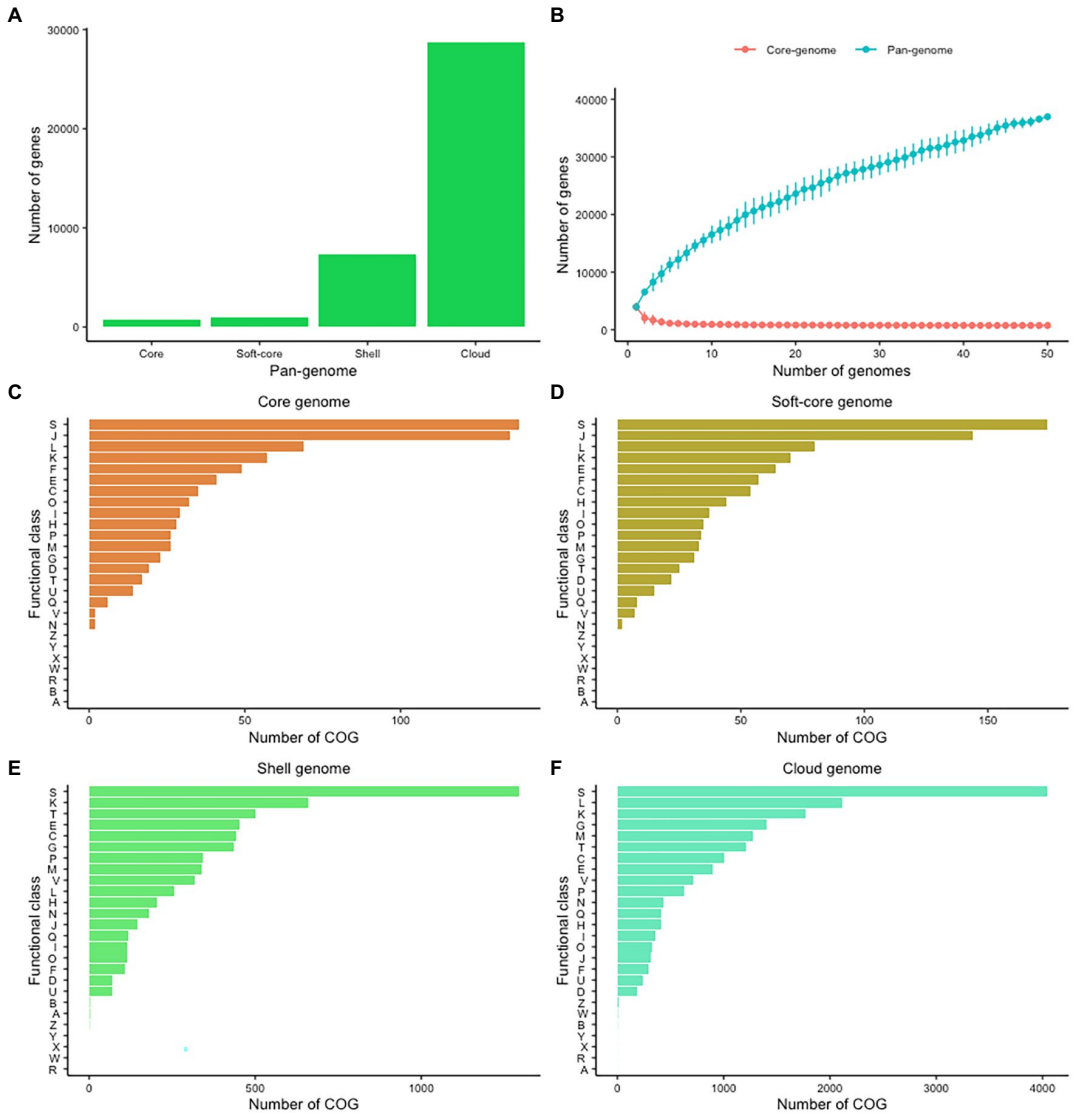
The pan-genome of CEC. **(A)** The sizes of core-, soft-core, shell-, and cloud-genomes at 80% BLAST cutoff identity. **(B)** The sizes of core- and pan-genomes in relation to numbers of genomes added into the gene pool as a function of the number of genomes included. **(C–F)** Distribution of cluster of orthologous genes (COGs) in the core, soft-core, shell-, and cloud-genomes. COG categories: A, RNA processing and modification; C, energy production and conversion; D, cell cycle control and mitosis; E, amino acid metabolism and transport; F, nucleotide metabolism and transport; G, carbohydrate metabolism and transport; H, coenzyme metabolism; I, lipid metabolism; J, translation; K, transcription; L, replication and repair; M, cell wall/membrane/envelop biogenesis; N, cell motility; O, post-translational modification, protein turnover, chaperone functions; P, inorganic ion transport and metabolism; Q, secondary structure; R, general functional prediction only; S, function unknown; T, signal transduction; U, intracellular, trafficking and secretion; V, defense mechanisms; W, extracellular structures; X, mobilome: prophages, transposons; and Z, cytoskeleton.

The variability of CEC genomes may have contributed to interspecies or intraspecies diversity, population genetics, and evolution. We therefore explored the entire genomic repertoire of the CEC by systematically determining its pan-genome with the BLAST identity cutoff at 60%, 70%, 80%, 90%, and 95%. The cutoff had a significant effect on the CEC pan-genome whereby its increase corresponded with a decrease in core- and soft-core genomes and an increase in shell- and cloud-genomes (Supplementary Table 3). Because of these effects, further pan-genome analyses were performed on the data set from 80% BLAST identity cutoff whereby the core- and soft-core genomes comprised of 748 and 942 genes, respectively, while the shell- and cloud-genomes had 7,296 and 28,741 genes, respectively (Figure 2A). The core genome represented 14.19%–22.40% of the gene content of each strain, while the cloud genes represented 77.72% of the pan-genome. The large accessory genome highly suggests a significant degree of genomic diversity within CEC. The pan-genome frequency showed a proportional relationship between the number of genes and genomes and the curve analysis indicates the CEC pan-genome is open (Figure 2B). The pan-genome of CEC had an alpha of 0.456. Similarly, the pan-genome of *C. estertheticum* had an alpha of 0.392. Given that the pan-genome is closed if the estimated alpha is above 1.0, both pan-genomes can be considered to be open.

### Evaluation of Cluster of Orthologous Groups Reveal Variability in Carbohydrate Metabolism and Transport in CEC

In a next step, we evaluated the cluster of orthologous groups (COGs) functional classification to define possible differences in the functions encoded by the core, soft-core, cloud-, and shell-genomes of CEC (Figures 2C–E). In all four cases, the highest proportion of genes were COG category S (Function unknown). Among the known functions, both core and soft-core gnomes consisted mostly of COG categories J (Translation), L (Replication and repair), K (Transcription), E (Amino Acid metabolism and transport), and F (Nucleotide metabolism and transport), which suggest that the processes controlling the flow of genetic information in CEC are conserved. On the other hand, the shell-genome was biased toward K (Transcription), T (Signal Transduction), E (Amino Acid metabolism and transport), C (Energy production and conversion), G (Carbohydrate metabolism and transport) while the cloud genome consisted mostly of L (Replication and repair), K (Transcription), M (Cell wall/membrane/envelop biogenesis), G (Carbohydrate metabolism and transport) and T (Signal Transduction) categories. The identification of carbohydrate metabolism and transport category in both cloud- and shell-genomes suggests that these processes are among the most genetically variable within CEC.

### Recombination Influences the Population Structure of CEC

**Figure 3:**
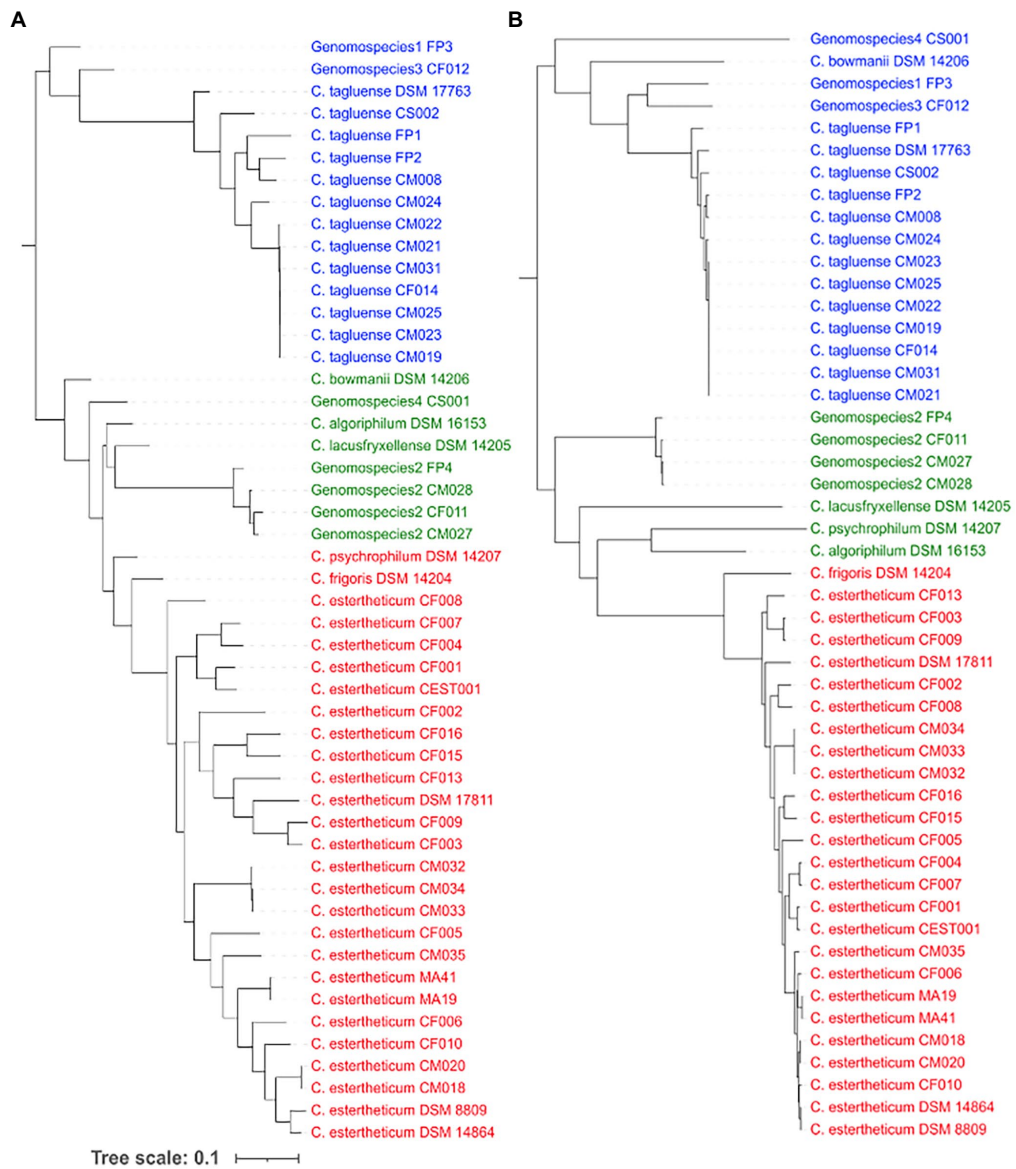
Influence of recombination events on the population structure of CEC. The core genome-based phylogeny of CEC **(A)** before and **(B)** after the removal of recombination events and three corresponding clades from each tree are highlighted in blue, green, and red fonts.

To determine whether recombination events have influenced the population structure of CEC, we first determined the occurrence of recombination within the core genome of CEC. The core-genome-based phylogenetic tree (Figure 3A) confirmed all species identified by the *rpoB* gene (Figure 1A), but the population structure varied slightly whereby it was divided into Clade 1 consisting of *C. estertheticum*, *C. frigoris*, and *C. psychrophilum*, Clade 2 consisting of *C. bowmanii C. algoriphilum C. lacusfryxellense* genomospecies2 and genomospecies4 and Clade 3 consisting of *C. tagluense*, genomospecies1, and genomospecies3. Within the core genome, we identified 252 recombination events, out of which 70 were ancestral, that is, they occurred between or among strains, while 182 were strain-specific (Supplementary Figure 5). Among, the ancestral recombination events, the majority occurred within the species and more specifically between strains that were more closely related than distantly related. The reconstruction of the core phylogenetic tree without the recombined regions (Figure 3B) revealed the influence of recombination on the population structure of CEC. While three clades were identified, the species within these clades varied slightly with those before the removal of recombination events. Clade 1 consisted of *C. estertheticum* and *C. frigoris*, Clade 2 consisted of *C. algoriphilum*, *C. lacusfryxellense,* and *C. psychrophilum* and genomspecies2 while Clade 3 consisted of *C. tagluense*, *C. bowmanii*, genomospecies1, genomospecies3, and genomospecies4. The data show that recombination events within CEC occur regularly and have also contributed to the current population structure of the complex.

### The Reduced Genome and Speciation of Genomospecies2 Correspond With Absence of Flagella Related Genes

**Figure 4:**
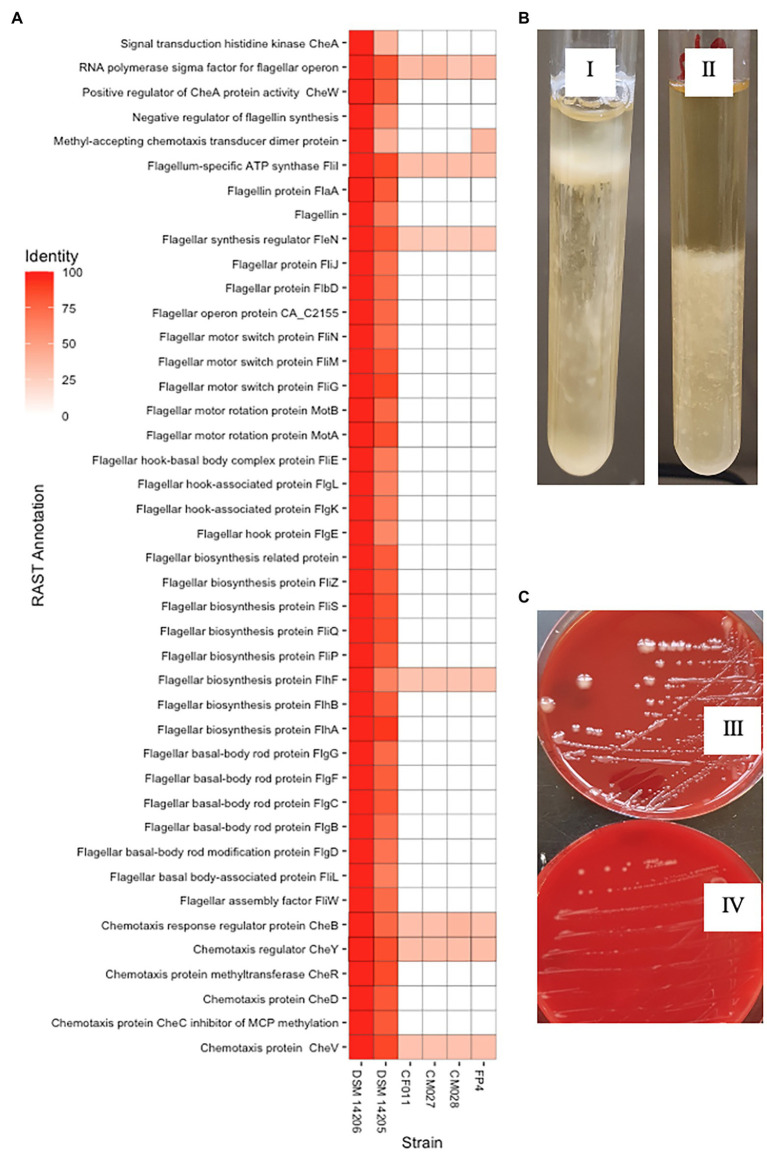
Unique genotypic and phenotypic characteristics of genomospecies2. **(A)** Whole-genome sequence-based comparison in RAST server of genomospecies2 strains FP4, CF011, CM027, and CM028 against *Clostridium bowmanii* DSM 14206 and *Clostridium lacusfryxellense* DSM 14205 reveals the lack of flagellar assembly and signaling genes in genomospecies2. **(B)** Motility test in Reinforced Clostridium Media broth shows genomospecies2 CF011 has reduced motility compared to *C. bowmanii* DSM 14206. Both strains were incubated for 14 days without shaking. **(C)** On Columbia Blood Agar plates, genomospecies2 CF011 forms larger and slimy colonies unlike *C. bowmanii* DSM 14206. Both strains were incubated for 8 weeks.

The variable interspecies genome sizes within CEC shows genes have been acquired or lost within the complex, which might contribute to speciation. This prompted us to determine signature genes that have been acquired or lost within CEC and determine whether the loss or gain may have contributed to speciation events within the complex. In this case, we investigated genomospecies2, which has the least genome size of less than 4.0 Mbp (Supplementary Figure 4). An analysis of the CEC accessory genome led to the identification of nine genes that were exclusively absent in genomospecies2. These included chemotaxis-related genes (*cheA*, *cheD*, and *cheW*), RNA polymerase sigma-D factor, and two putative type III secretion system genes that were annotated as putative ATP synthase YscN and Yop proteins translocation protein U. Further analysis of the genes showed each CEC strain had 23–28 genes related to chemotaxis and flagella assembly while genomospecies2 strains had one gene each (data not shown). A whole-genome sequence-based comparison of genomospecies2 and the two closely related species, *C. bowmanii* and *C. lacusfryxellense* using RAST annotation server showed up to 55 related genes were absent in genomospecies2 (Figure 4A) further confirming the absence of these genes. The lack of these genes was consistent with reduced motility of the genomospecies2 CF011 when compared with *C. bowmanii* DSM 14206 in RCM media (Figure 4B). An investigation of genomospecies2 strains CF011, CM027, and CM028 colony morphology showed they form significantly larger and slimy colonies after extended incubation periods (>8 weeks) compared with all other CEC species (Figure 4C). Further analysis of gene presence/absence in the pan-genome matrix did not reveal any exopolysaccharide genes that were specific to genomospecies2. Therefore, genomospecies2 is currently the only known CEC species lacking genes related to flagella signaling and assembly and this loss influences the motility and may also be linked with the unique colony morphology.

### Identification of Conserved Metabolic Genes That Are Present/Absent in Two CEC Phylogroups

Although CEC causes meat spoilage, the spoilage is distinguished as either corresponding with formation of copious or low amounts of gas (Table 1). This prompted us to investigate the presence/absence of conserved metabolic genes that can explain these differences using *C. estertheticum* and *C. tagluense* as representative species. The analysis of the CEC accessory genome revealed 166 and 412 genes that were only present in *C. estertheticum* and *C. tagluense*, but no obvious genes could be linked to meat spoilage that is characteristic of the two species or CEC in general (data not shown). Therefore, we hypothesized that these metabolic genes are also conserved with other species given the high relatedness of closely related CEC species. Therefore, the CEC species were divided into two major phylogroups, phylogroup 1 (PG1) and Phylogroup 2 (PG2), which is consistent with the *rpoB* gene phylogenetic tree. PG1 consisted of *C. estertheticum*, *C. frigoris*, *C. psychrophilum*, and *C. algoriphilum* while PG2 consisted of *C. tagluense*, *C. lacusfryxellense*, *C. bowmanii*, genomospecies1, genomospecies2, genomospecies3, and genomospecies4. The analysis identified 63 and 27 genes that were conserved in PG1 and PG2, respectively. Elimination of seven genes with similar annotations between the two groups and 12 hypothetical proteins allowed 71 (49 and 14 genes in PG1 and PG2, respectively) genes to be compared. Interestingly, genes linked to hydrogen and carbon dioxide production could be identified among the 71 genes. Among these included formate hydrogenlyase subunits 4 and 7 and Hydrogenase-4 components B and G that were specific to PG1 while hydrogenase isoenzymes formation protein HypE was specific to PG2. This suggested that PG1 and PG2 have evolved different fermentation pathways that might influence gas production. Further comparison of the 71 genes revealed pentose metabolic genes (arabinose metabolism transcriptional repressor, arabinose operon regulatory protein, arabinose-proton symporter L-arabinose isomerase, L-ribulose-5-phosphate 4-epimerase, ribulokinase, and transaldolase) and lactose and galactose metabolic genes (lactose permease and tagatose-6-phosphate kinase) were identified in PG1 only. This supports the COG analysis and further suggests PG1 and PG2 have evolved different carbohydrate metabolic pathways which might not only influence their habitat adaptation, but also meat substrate utilization and their respective meat spoilage mechanisms.

### Variable Utilization of Carbohydrates Is Linked With Variable Distribution of Carbohydrate-Active Enzymes in *Clostridium estertheticum* and *Clostridium tagluense*

**Figure 5:**
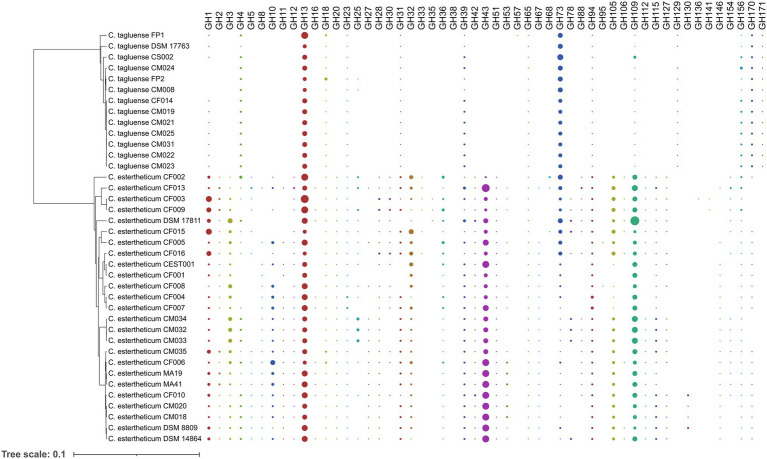
Distribution of glycoside hydrolases in *C. estertheticum* and *C. tagluense*. The size of each circle corresponds with the frequency of the gene. A blank shows the gene is absent. The phylogeny is based on the *rpoB* gene.

To determine the differences in carbohydrate metabolic pathways between PG1 and PG2, we specifically investigated the distribution of six groups of carbohydrate-active enzymes (CAZy), namely, carbohydrate esterases (CE), carbohydrate-binding modules (CBM), glycoside hydrolases (GH) glycosyltransferases (GT), polysaccharide lyases (PL), and auxiliary activities (AA), in *C. estertheticum* and *C. tagluense* genomes (Supplementary Figure 6), which represented PG1 and PG2, respectively. In *C. estertheticum*, GHs were the most frequent CAZy genes and ranged from 40 to 69 per genome. In contrast, GTs, which ranged from 30 to 42 per genome, were the most frequent in *C. tagluense*. Overall, the frequencies of AAs, PLs, CBMs, GTs, and GHs were higher (*p* < 0.05) in *C. estertheticum* than in *C. tagluense*. However, GTs were more frequent (*p* < 0.05) in *C. tagluense* than in *C. estertheticum*. An in-depth analysis of the GHs revealed the two species encoded 53 different types of enzymes (Figure 5), with *C. estertheticum* having the highest diversity of the enzymes. Specifically, 36 GHs were only identified in *C. estertheticum* and eight of these GHs (GH3, GH20, GH32, GH36, GH42, GH43, GH51, and GH115) were present in all 25 *C. estertheticum* genomes while two GHs (GH28 and GH105) were present in 24 *C. estertheticum* genomes. In contrast, only three GHs were exclusively identified in *C. tagluense*. Among these, GH171 was present in all *C. tagluense* genomes while GH129 was absent in only one genome. Despite the differences, nine GHs (GH13, GH18, GH23, GH39, GH65, GH73, GH94, GH109, and GH156) were present in all *C. estertheticum* and *C. tagluense* genomes. Among these, GH13, GH94, and GH109 were significantly higher (*p* < 0.05) in *C. estertheticum* than in *C. tagluense* while GH73, and GH156 were significantly higher (*p* < 0.05) in *C. tagluense* than in *C. estertheticum* (Supplementary Figure 7). Therefore, this confirmand the variable distribution of carbohydrate utilization genes between *C. estertheticum* and *C. tagluense*.

The variable distribution of carbohydrate utilization genes between the two species suggested the range of substrates they can utilize also differs. To validate this, we determined the utilization of 16 substrates (Table 2). *Clostridium estertheticum* strains utilized D-glucose, D-lactose, D-sucrose, D-maltose, D-salicin, D-xylose, L-arabinose, D-cellobiose, and D-raffinose. The strains showed variable utilization of six other substrates and did not utilize D-trehalose. Comparatively, *C. tagluense* strains utilized only three substrates, D-glucose, D-maltose, and D-trehalose while three strains utilized glycerol. Therefore, the high number of GHs in *C. estertheticum* corresponded to utilization of diverse fermentable substrates compared to *C. tagluense*.

**Table 2:** Substrate utilization by in-house *C. estertheticum* and *Clostridium tagluense* (+, positive reaction and −, negative reaction).

Strain ID	GLU	MAN	LAC	SAC	MAL	SAL	XYL	ARA	GLY	CEL	MNE	MLZ	RAF	SOR	RHA	TRE
*C. tagluense* CM008	+	−	−	−	+	−	−	−	+	−	−	−	−	−	−	+
*C. tagluense* CM019	+	−	−	−	+	−	−	−	−	−	−	−	−	−	−	+
*C. tagluense* CM021	+	−	−	−	+	−	−	−	−	−	−	−	−	−	−	+
*C. tagluense* CM022	+	−	−	−	+	−	−	−	−	−	−	−	−	−	−	+
*C. tagluense* CM023	+	−	−	−	+	−	−	−	−	−	−	−	−	−	−	+
*C. tagluense* CM024	+	−	−	−	+	−	−	−	−	−	−	−	−	−	−	+
*C. tagluense* CM025	+	−	−	−	+	−	−	−	−	−	−	−	−	−	−	+
*C. tagluense* CM031	+	−	−	−	+	−	−	−	−	−	−	−	−	−	−	+
*C. tagluense* CF014	+	−	−	−	+	−	−	−	−	−	−	−	−	−	−	+
*C. tagluense* CS002	+	−	−	−	+	−	−	−	+	−	−	−	−	−	−	+
*C. estertheticum* CEST001	+	+	+	+	+	+	+	+	−	+	+	−	+	−	−	−
*C. estertheticum* CF001	+	+	+	+	+	+	+	+	−	+	+	−	+	−	−	−
*C. estertheticum* CF002	+	+	+	+	+	+	+	+	+	+	+	−	+	+	+	−
*C. estertheticum* CF003	+	−	+	+	+	+	+	+	−	+	−	−	+	−	+	−
*C. estertheticum* CF004	+	+	+	+	+	+	+	+	−	+	+	−	+	+	−	−
*C. estertheticum* CF005	+	+	+	+	+	+	+	+	−	+	+	−	+	+	+	−
*C. estertheticum* CF006	+	+	+	+	+	+	+	+	−	+	+	−	+	+	+	−
C*. estertheticum* CF007	+	+	+	+	+	+	+	+	−	+	+	−	+	−	−	−
*C. estertheticum* CF008	+	−	+	+	+	+	+	+	+	+	+	−	+	−	−	−
*C. estertheticum* CF009	+	−	+	+	+	+	+	+	−	+	−	−	+	−	+	−
*C. estertheticum* CF010	+	+	+	+	+	+	+	+	−	+	+	−	+	+	+	−
*C. estertheticum* CF013	+	−	+	+	+	+	+	+	−	+	−	−	+	−	+	−
*C. estertheticum* CF015	+	+	+	+	+	+	+	+	−	+	+	−	+	+	+	−
*C. estertheticum* CF016	+	+	+	+	+	+	+	+	−	+	+	−	+	+	+	−
*C. estertheticum* CM018	+	+	+	+	+	+	+	+	−	+	+	−	+	+	+	−
*C. estertheticum* CM020	+	+	+	+	+	+	+	+	−	+	+	−	+	+	+	−
*C. estertheticum* CM032	+	+	+	+	+	+	+	+	−	+	+	−	+	+	+	−
*C. estertheticum* CM033	+	+	+	+	+	+	+	+	−	+	+	−	+	+	+	−
*C. estertheticum* CM034	+	+	+	+	+	+	+	+	−	+	+	−	+	+	+	−
*C. estertheticum* CM035	+	+	+	+	+	+	+	+	−	+	+	−	+	+	+	−
*C. estertheticum* DSM 14864	+	+	+	+	+	+	+	+	+	+	+	−	+	+	+	−

GLU, glucose; MAN, mannose; LAC, lactose; SAC, sucrose; MAL, maltose; SAL, salicin; XYL, xylose; ARA, arabinose; GLY, glycerol; CEL, cellobiose; MNE, mannose; MLZ, melezitose; RAF, raffinose; SOR, sorbitol; RHA, rhamnose; and TRE, trehalose.

### Variable Gas Production in Vacuum-Packed Meat by *Clostridium estertheticum* and *Clostridium tagluense* Corresponds With the Distribution of [NiFe] Hydrogenase *hyp* Gene Cluster

**Figure 6:**
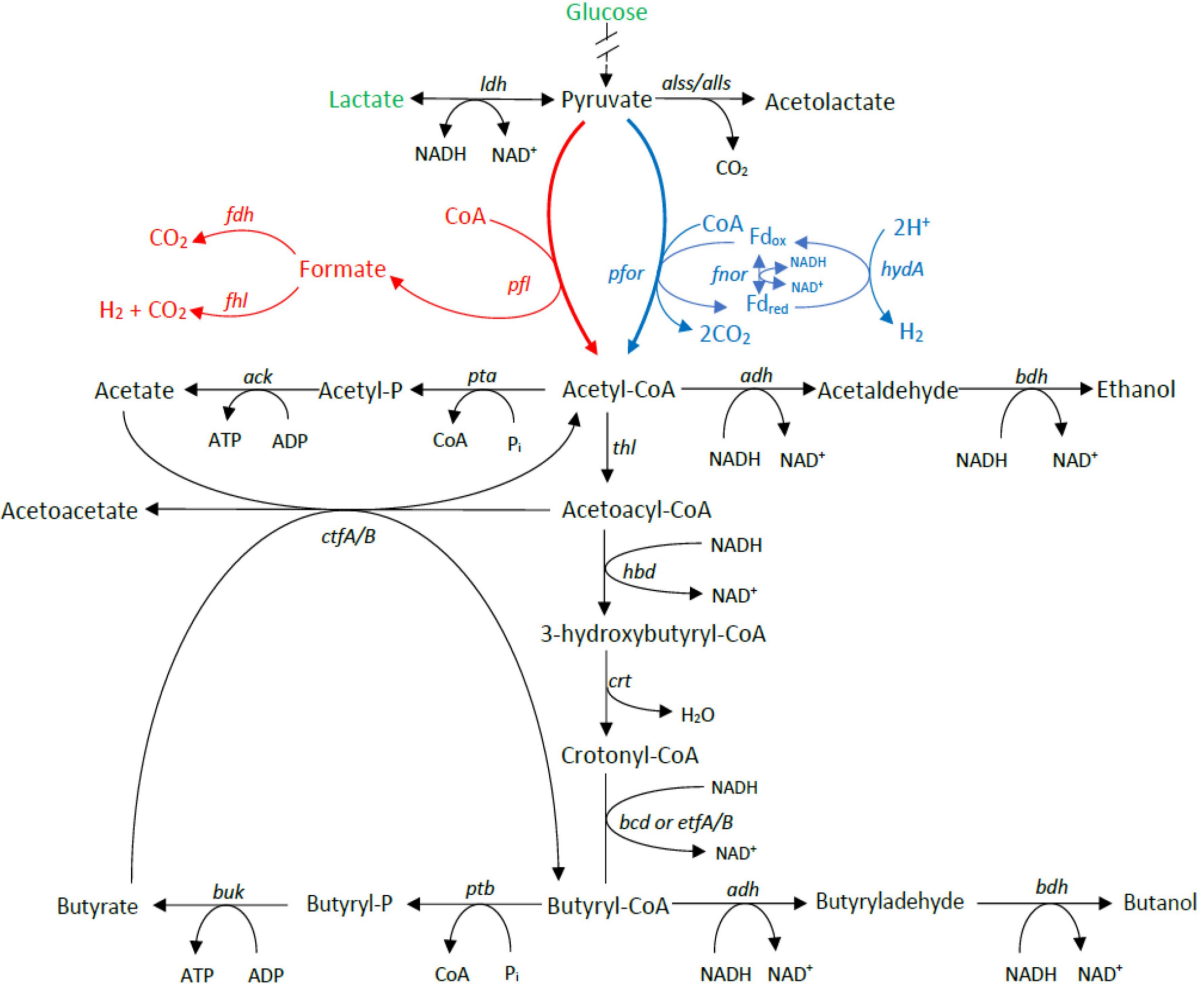
Genome-based reconstruction of *C. estertheticum* and *C. tagluense* central fermentation pathway. Two key pathways involved in gas production are highlighted in red or blue colors. The main substrates utilized by both species in meat, glucose, and lactate are highlighted in green. *ldh*, L-lactate dehydrogenase; *alls* and *alss*, acetolactate synthase large and small subunits; *pfor*, NADP^+^ pyruvate ferrodoxin oxidoreductase (*fnor-nfn*); *pta*, phosphate acetyltransferase; *ack*, acetate kinase; *adh*, alcohol dehydrogenase; *thl*, thiolase; *hbd*, hydroxybutyryl-coA dehydrogenase; *crt*, crotonase; *bcd/etfAB*, butyryl-CoA dehydrogenase/electron transfer flavoprotein NAD^+^ ferredoxin, subunits alpha and beta; *ptb*, phosphotransbutyrylase; *buk*, butyrate kinase; *ctfA/B*, coA-transferase subunits A/B; *bdh*, butanol dehydrogenase; *pfl*, pyruvate formate lyase; and *hydA*, hydrogenase A.

**Figure 7:**
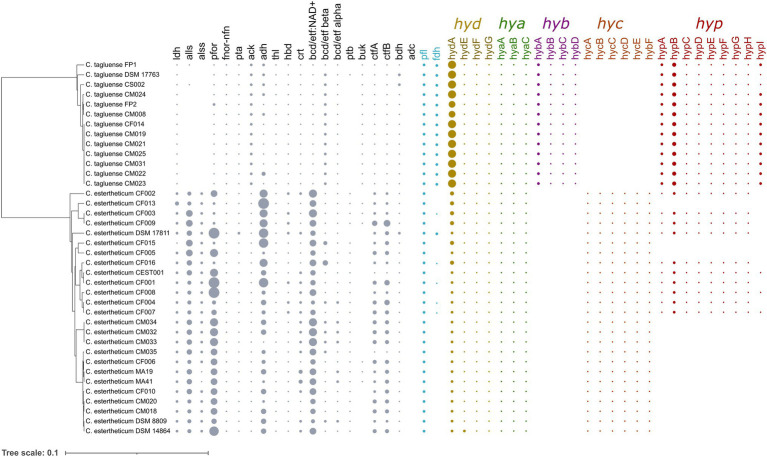
Distribution of acetone–butyrate–ethanol fermentation pathway and hydrogenases genes within *C. estertheticum* and *C. tagluense*. The five hydrogenase gene clusters, *hyd*, *hya*, *hyb*, *hyc*, and *hyp* are highlighted in different colors. The size of each circle corresponds with the frequency of the gene. A blank shows the gene is absent. The phylogeny is based on the *rpoB* gene. *ldh*, L-lactate dehydrogenase; *alls* and *alss*, acetolactate synthase large and small subunits; *pfor*, NADP^+^ pyruvate ferrodoxin oxidoreductase (*fnor-nfn*); *pta*, phosphate acetyltransferase; *ack*, acetate kinase; *adh*, alcohol dehydrogenase; *thl*, thiolase; *hbd*, hydroxybutyryl-coA dehydrogenase; *crt*, crotonase; *bcd/etfAB*, butyryl-CoA dehydrogenase/electron transfer flavoprotein NAD^+^ ferredoxin, subunits alpha and beta; *ptb*, phosphotransbutyrylase; *buk*, butyrate kinase; *ctfA/B*, coA-transferase subunits A/B; *bdh*, butanol dehydrogenase; *adc*, acetone dehydrogenase; *pfl*, pyruvate formate lyase; and *hyd*, *hya*, *hyb*, *hyc*, and *hyp*, hydrogenase gene clusters.

Carbohydrate utilization is liked with gas production in *Clostridium* genus through the acetone–butanol–ethanol fermentation pathway. To determine whether the differences in gas evolution during meat spoilage between the *C. estertheticum* and *C. tagluense* is solely linked with differences in the carbohydrate utilization genes or there exists other underlaying genetic influences, we first reconstructed the acetone–butyrate–ethanol fermentation pathway to include steps for the production of lactate, formate, ethanol, acetate, butyrate, acetone, acetoin, and butanol (Figure 6). The genes involved in the pathway were present in both species with minor variations including copy numbers (Figure 7). This suggested the central acetone–butyrate–ethanol fermentation pathway was conserved between the two species at the genetic level and cannot fully explain the differences in gas production. We therefore carried in-depth analysis whereby we identified and compared two different processes that produce H_2_ and CO_2_ through the oxidation of pyruvate in the acetone–butyrate–ethanol fermentation pathway (Figure 6). The first process involves the oxidative decarboxylation of pyruvate to acetyl-coA and CO_2_ by pyruvate ferrodoxin oxidoreductase (PFOR). This process requires the reduction of ferredoxin, which is further oxidized simultaneously by [FeFe] hydrogenase, HydA, and ferredoxin NADH oxidoreductase (FNOR) resulting in the formation of H_2_ (Figure 6). All genes involved in this process including *pfor*, *fnor*, and *hydA* were present in both species although the copy number of *hydA* in *C. tagluense* was higher than in *C. estertheticum* (Figure 7). The genes involved in the maturation of HydA, that is *hydE*, *hydF*, and *hydG*, were also identified (Figure 7). The second process involves the decarboxylation of pyruvate to acetyl-coA and formate by pyruvate formate lyase (PFL; Figure 6). The formate is then oxidized to CO_2_ by a formate dehydrogenase (Fdh) or to CO_2_ and H_2_ by formate-hydrogenase lyase complex (FHL; comprising of Fdh and a hydrogenase; Figure 6). The presence of the *pfl* gene indicated the process of formate production is conserved in both species (Figure 7). However, the *fdh* gene was present in all *C. tagluense* genomes and six *C. estertheticum* genomes (Figure 7).

Interestingly, we identified other gene clusters, which likely encode putative hydrogenases that can play an important role in production of molecular hydrogen. A gene cluster for bifurcating [FeFe] hydrogenase encoding genes for *alpha*, *beta,* and *gamma* subunits was identified in both species. The bifurcating [FeFe] hydrogenase is herein referred to as Hya and is encoded by the *hya* cluster consisting of *hyaABC* genes, encoding the alpha, beta, and gamma subunits, respectively (Figure 7). The presence of *hyaABC* genes suggests the metabolic process of its product is conserved in both species and together with HydA hydrogenase may also not fully explain the differences observed in gas production at the genetic level. Additionally, we found three different hydrogenase gene clusters in both species (Figure 7). One cluster herein referred to as *hyc* cluster consists of six genes *hycBCDEFG* was exclusive to *C. estertheticum*. The second cluster, herein referred to as *hyp* cluster consisting of nine [NiFe] hydrogenase genes in the order *hypABCDEFGHB* was present in all *C. tagluense* strains and nine *C. estertheticum* strains. The strains with the *hyp* cluster formed two distinct clusters within *C. estertheticum* suggesting the intraspecies presence or absence of the cluster was evolutionary. The third [FeFe] hydrogenase gene cluster, here in referred to as *hyb* cluster consisted of four genes *hybABCD*, and was exclusive to *C. tagluense*. We speculated the variable distribution of the *hyb*, *hyc,* and *hyp* hydrogenase gene clusters could explain the phenotypic differences in gas production in meat between *C. estertheticum* and *C. tagluense*.

**Figure 8:**
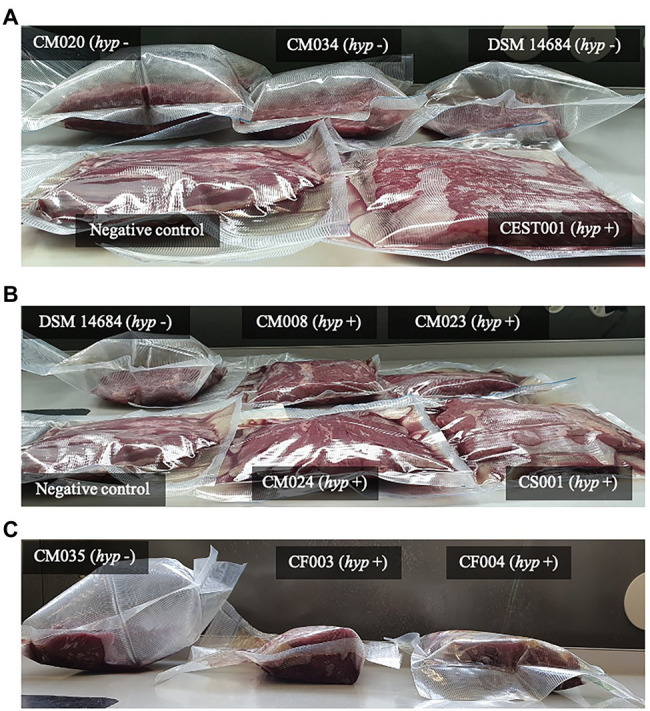
Gas production in vacuum-packed beef at 8°C by *C. estertheticum* and *C. tagluense* with (*hyp* +) or without (*hyp* −) *hyp* hydrogenase gene cluster. **(A)** The *hyp* − *C. estertheticum* CM020, CM034, and DSM 14864 caused pack distention while the *hyp* + *C. estertheticum* CEST001 only caused loss of vacuum. **(B)** The *hyp* + *C. tagluense* CM008, CM023, CM024, and CS002 caused loss of vacuum. *Clostridium estertheticum* DSM 14864 (*hyp* −) was the positive control. **(C)** The *hyp* + *C. estertheticum* CF003 and CF004 did not cause pack distention even after 56 days of incubation compared to the *hyp* − *C. estertheticum* CM035. In **(A)** and **(B)**, the samples were incubated for 28 days.

To link the genome and phenotype, we tested gas production by both species in meat. Three out of the four *C. estertheticum* strains DSM 14864, CM020, and CM034 caused pack distention while one *C. estertheticum* strain, CEST001, and all four *C. tagluense* strains CM008, CM023, CM024, and CS002, did not cause pack distention (Figures 8A,B). This was surprising since variable differences in pack distention by *C. estertheticum* have not been reported before. At the genomic level, the four *C. estertheticum* strains had *hyd*, *hya,* and *hyc* gene clusters while all four *C. tagluense* strains had *hyd*, *hya*, *hyb*, and *hyp* genes. *Clostridium estertheticum* CEST001, which did not cause pack distention also had the *hyp* genes, which led to the suggestion that the presence of the *hyp* gene cluster was linked with absence of pack distention. Further analysis of selected *C. estertheticum* strains with or without the *hyp* genes consistently showed *C. estertheticum* CM035 (*hyp* −) caused pack distention while *C. estertheticum* CF003 and CF007 (*hyp* +) did not cause pack distention despite extended incubation periods of 56 days (Figure 8C). Therefore, the differences in the intra- and interspecies distribution of hydrogenases gene clusters, and specifically the *hyp* cluster, may be associated with meat spoilage with or without gas production by *C. estertheticum* and *C. tagluense* species.

### BPS Causing Clades of *Clostridium estertheticum* Possess Unique Spore Germination Genes

We finally took advantage of the large repertoire of *C. estertheticum* genomes (*n* = 25) to perform an intraspecies comparative genomics. Using a 80% BLAST identity cutoff, we showed *C. estertheticum* has a pan-genome of 20,111 genes. The core- and soft-core genomes comprised of 1,771 and 2,036 genes, respectively, while the shell- and cloud-genomes had 4,354 and 13,721 genes, respectively. The large accessory genome highly suggests a significant degree of genomic diversity within *C. estertheticum*.

We further created a maximum likelihood phylogenetic tree which revealed six *C. estertheticum* putative clades (Supplementary Figure 8). Interestingly, the *C. estertheticum* reference strains known to cause BPS, DSM 8809, DSM 14864, MA19, and MA41 and the BPS causing strains in the present study, CM020 and CM035 clustered together in Clade 5 (Supplementary Figure 8), which is *hyp* − (Figure 7). The three strains causing BPS cases in Switzerland CM032, CM033, and CM034 clustered in Clade 3, which is also *hyp* − (Figure 7). Because of the putative clades’ relevance to BPS, we determined the presence of other important genes that are uniquely specific to these clades. Four genes annotated as “putative response regulatory protein,” “Spore germination protein B2,” “Spore germination protein B1,” and “DegV domain-containing protein” were identified. The identification of the two spore germination proteins genes that are specific to these two putative clades suggests they have evolved different spore germination pathways.

## Discussion

CEC includes diverse psychrophilic and anaerobic species that are commonly isolated from the meat processing environment, and are collectively recognized as a major cause of meat spoilage in chilled and vacuum-packed meat with *C. estertheticum* being regarded as the most important among the named CEC species (Wambui and Stephan, 2019). Previously, *C. estertheticum* has been reported to cause BPS in a diverse range of meat including lamb, beef and pork (Collins, 1992; Byrne et al., 2009; Zhang et al., 2020) but for the first time, we have shown here that it can also cause spoilage in horse meat (Figure 1C). Interestingly, we noted that *C. estertheticum* CM032, which caused BPS in beef, and CM033 and CM034, which caused BPS in horse meat samples, were clonal (Supplementary Figure 3). This is also the first reported case of a *C. estertheticum* clone causing meat spoilage in two different types of meat and suggests a similar source of contamination. Therefore, this study not only demonstrates the suitability of whole-genome sequencing in the identification of CEC strains, but also determination of the relatedness of strains causing meat spoilage thus facilitating traceability and inference of the source of contamination.

So far, 10 CEC species had been identified (Palevich et al., 2021; Wambui et al., 2021a). The identification of strain CS001 which forms the novel species, genomospcies4 (Figures 1A,B), has expanded the number of species to 11 further revealing the diversity of CEC. Interestingly, strain CS001 forms a divergent branch within CEC (Figures 1A,B) suggesting the probable existence of yet unsampled intermediate CEC species between genomspecies4 and other CEC species. The fact that the strain CS001 from the novel genomospecies4 species and other recently described species (Table 1) could also cause meat spoilage, further reinforces CEC’s role in the spoilage of chilled vacuum-packed meat.

Difficulties in isolation and culturing of CEC strains have continuously limited the number of CEC genomes available for the inter- and intraspecies genomic characterization. This is evident in a previous study where only six strains from four CEC species were genomically characterized (Palevich et al., 2021). Accordingly, our work has not only increased the availability of CEC genomes, but has gone ahead to perform a comprehensive comparative genomic analysis that has revealed the main forces underpinning the genetic diversity and evolution of 11 CEC species. The analysis of recombination events has shown that although recombination is limited to closely related strains, the events are high and have an influence on the population structure of CEC, which was demonstrated by the reconstruction of the core-genome-based phylogeny of CEC before and after removal of recombined regions (Figure 3). This is consistent with a previous report that recombination can influence the bacterial phylogeny (van Rossum et al., 2020; Lin et al., 2021). Although we have identified recombination as one of the drivers of genetic diversity within CEC, gene gain and loss events are also recognized as major drivers for genetic diversity and result in the evolution of bacteria with unique genotypes and phenotypes (Li et al., 2017; van Rossum et al., 2020). In this regard, we have identified several candidate species-specific genes that have been lost by genomospecies2 and likely contributed to its reduced genome size and speciation. Among these genes were the flagellar assembly and signaling genes (Figure 4A). Flagella are particularly important for bacteria motility (Moens and Vanderleyden, 1996). Consistently, the absence of the genes corresponded with reduced motility of genomospecies2 strains (Figure 4B). The current analysis was carried out in broth culture, which might indicate swimming motility of genomospecies2 strains in liquid media is impaired. Further motility studies including swarming motility, which determines the movement of bacteria across a surface with the aid of flagella are proposed. We also noted that the absence of these genes corresponded with formation of larger and slimy colonies by genomospecies2 compared to other CEC species (Figure 4C) suggesting altered colony morphology and production of extracellular matrix. Similar results have been reported in *Vibrio cholerae* and it has been suggested that the absence of flagella constitutes a signal to increase exopolysaccharide synthesis (Watnick et al., 2001). Further studies will be required to identify genes responsible for the synthesis of the matrix by genomospecies2 and determination of its composition.

By exploiting the pan-genome of CEC, its genetic diversity was further unraveled and attributed to the large accessory genome. Accessory genes are important for fundamental bacterial functions, but can also determine the diversity of bacteria (Adeniji et al., 2019). Through the analysis of cluster of orthologous groups, carbohydrate metabolism and transport emerged as one of the key functions of the accessory genome (Figures 2E,F) while the analysis of gene presence/absence showed that the two CEC phylogroups differed with carbohydrate metabolism genes indicating they have evolved different saccharolytic abilities. This is particularly interesting because several studies have reported differences in fermentable substrate utilization among CEC members (Spring, 2003; Suetin et al., 2009; Yang and Badoni, 2013). In the current study, we have used a diverse range of strains from *C. estertheticum* and *C. tagluense* and confirmed previous reports that utilization of fermentable substrates by *C. estertheticum* is higher than in *C. tagluense* (Suetin et al., 2009). Using a higher repertoire of CEC strains and genomes than any previous study, we showed the phenotypes (Table 2) were consistent with the distribution of carbohydrate utilization genes (Figure 5) in both species. These data suggest *C. estertheticum* is highly adaptable to environment containing diverse carbohydrate substrates.

With respect to meat spoilage, the utilization of intramuscular carbohydrates by members of CEC has been reported to be an important link to the acetone–butyrate–ethanol fermentation pathway, which ultimately results in meat spoilage (Palevich et al., 2021). In the current study, we have gone ahead and determined the genetic basis for differences in gas production during meat spoilage by CEC using *C. estertheticum* and *C. tagluense* as representative species. While meat spoilage accompanied by gas production by *C. estertheticum* could have been as a consequence of higher number of carbohydrates metabolizing genes (Figure 5), which corresponded with high range of substrate utilization compared to *C. tagluense* (Table 2), the revelation that some *C. estertheticum* strains also caused meat spoilage with less gas production (Figures 8A,C) did not fully support this hypothesis. Further analyses have however linked the gas production to the inter-genomic distribution of hydrogenases (Figure 7). Hydrogenases (H_2_ases) are redox metalloenzymes that catalyze the reversible oxidation of molecular hydrogen (H_2_; Schuchmann et al., 2018). Anaerobic bacteria, such as *Clostridium* spp., produce H_2_ after the reduction of protons by H_2_ases as means of disposing excess reducing equivalents while H_2_ produced from other metabolic pathways can be recycled by uptake H_2_ases (Vignais et al., 2001). Based on their metal content, there are three types of hydrogenase enzymes: mononuclear [Fe] and dinuclear [FeFe] and [NiFe] (Kaur-Ghumaan and Stein, 2014). Accordingly, we have shown that the [FeFe] and [NiFe] hydrogenases are encoded in the genomes of *C. estertheticum* and *C. tagluense* (Figure 6; Supplementary Table 2). The *hya* and *hyb* gene clusters encode [FeFe]-H_2_ases while the *hyc* and *hyp* gene clusters encode [NiFe]-H_2_ases gene clusters with each cluster consisting of a repertoire of genes necessary for the assembly and maturation a functional hydrogenases. These include genes necessary for catalytic activity of the hydrogenase, proteases involved in formation or maturation of subunits and membrane proteins involved in membrane binding (Trchounian et al., 2012).

The intragenomic presence of different [FeFe]-H_2_ase and [FeFe]-H_2_ase gene clusters in both *C. estertheticum* and *C. tagluense* suggest functional redundance of each type of hydrogenase. In *Clostridium* spp., this allows strains to adopt a wide range of environmental conditions (Calusinska et al., 2010). The [FeFe]-H_2_ases are typically associated with H_2_ production while [NiFe]-H_2_ases are mainly associated with H_2_ uptake (Peters et al., 2015). Interestingly, we have shown the presence *hyp* gene cluster, which encodes a putative [NiFe]-H_2_ase corresponds with absence of pack distention in both *C. estertheticum* and *C. tagluense* (Figure 8). Similar to other characterized homologous *hyp* gene clusters (Buhrke et al., 2001), we hypothesize that the gene products of *C. estertheticum* and *C. tagluense hyp* gene clusters are responsible for the assembly and maturation of hydrogen oxidizing [NiFe]-H_2_ases. Its absence in strains causing pack distention, such as *C. estertheticum* CM034 and DSM 14864 (Figure 8A), suggests these strains are incapable of reincorporating the hydrogen that is produced as a metabolite hence resulting in the accumulation and hence the phenotype that is characteristic of BPS. On the other hand, the strains with the *hyp* gene cluster, such as *C. estertheticum* CEST001 (Figure 8A), CF003, and CF004 (Figure 8C) and *C. tagluense* CM008, CM023, and CM024 (Figure 8B), are capable of recycling the hydrogen which prevents its accumulation in the packs hence lack of pack distention. The current lack of genetic tools amenable to CEC and the slow growth rates of CEC strains are a major limitation to the validation of these hypotheses through mutagenesis. Despite of these limitations, our study has provided a basis for future characterization of the *hyp* gene cluster.

The availability of 25 *C. estertheticum* genomes in the current study could provide some insights into the population structure and genetic diversity of the species. The pan-genome analysis demonstrated high genetic diversity within the species. Similarly, we identified six putative clades of the species (Supplementary Figure 8), two of which consist of strains known to cause BPS. Through intraspecies genomic comparison of *C. estertheticum* we identified that these two putative clades, which consist of *hyp* − strains, possess unique spore germination genes. Spore germination is critical process for the outgrowth and development of spoilage potential of sporulating bacteria (Delbrück et al., 2021). Therefore, further determination of spore germination process of the *hyp* − strains is necessary. Due to the limited number of genomes of the species, these clades are postulated to be putative and the current intraspecies comparative genomics are preliminary. More genomes are required for robust analysis to determine the most probable population structure and intraspecies genetic diversity of the species.

## Conclusion

This study provides a comprehensive genomic analysis of the *C. estertheticum* complex (CEC), a diverse group of psychrophilic bacteria associated with the spoilage of chilled vacuum-packed meat. Following the isolation, identification, and sequencing of 15 new CEC strains, we have expanded the number of CEC species to 11 and the number of publicly available genomes to 50. Presently, we have shown that recombination and gene gain/loss events are important sources of natural variation within CEC. Accordingly, genomospecies2 has lost genes related to flagellar assembly and signaling, which influences its motility and colony morphology. Through pan-genome analysis and phenotypic validation using the *C. estertheticum* and *C. tagluense* as representative species, we have shown the variable carbohydrate utilization within CEC is linked with variable distribution of carbohydrate-active enzymes, and more specifically, the glycoside hydrolases. Our findings that some *C. estertheticum* strains caused meat spoilage without pack distention suggest that these differences do not fully account for differences in gas production in vacuum-packed meat by *C. estertheticum* and *C. tagluense*. However, an important finding of our study is the revelation that the inter- and intraspecies differences exhibited by the two species during meat spoilage are associated with the distribution of the [NiFe]-hydrogenase *hyp* gene cluster. The absence of the cluster is associated with pack distention and its presence is associated with lack of pack distention suggesting the hydrogenase catalyzes the oxidation of molecular hydrogen preventing its accumulation in vacuum packs. We propose mutagenesis of the [NiFe]-hydrogenase *hyp* gene cluster be carried out in future.

## Data Availability Statement

The datasets presented in this study can be found in online repositories. The names of the repository/repositories and accession number(s) can be found in the article/Supplementary Material.

## Author Contributions

JW and RS designed the study. MS and NC carried out whole-genome sequencing and assembly. JW and MS carried out the bioinformatic analysis. JW carried out the phenotypic tests and wrote the initial draft manuscript. JW, MS, NC, and RS revised the final manuscript. RS supervised the study. All authors contributed to the article and approved the submitted version.

## Conflict of Interest

The authors declare that the research was conducted in the absence of any commercial or financial relationships that could be construed as a potential conflict of interest.

## Publisher’s Note

All claims expressed in this article are solely those of the authors and do not necessarily represent those of their affiliated organizations, or those of the publisher, the editors and the reviewers. Any product that may be evaluated in this article, or claim that may be made by its manufacturer, is not guaranteed or endorsed by the publisher.

## Footnotes

^1^ https://github.com/tseemann/shovill
