# Supplementary material for: Unraveling the Genotypic and Phenotypic Diversity of the Psychrophilic Clostridium estertheticum Complex, a Meat Spoilage Agent
Source: Front Microbiol. 2022 Mar 28;13:856810. doi: 10.3389/fmicb.2022.856810 (PMC8996182; doi:10.3389/fmicb.2022.856810)
Supplement: Supplementary file 1 [file Table_1.docx]

Supplementary Material

**Supplementary Table 1**. Description of 50 *Clostridium estertheticum* complex genomes used in the current study. (Genomes sequenced (n=16) in the current study are highlighted in red font => additional genomes analyzed in the current study, which has also been published previously by Wambui et al., 2022)

| Species | Strain ID | Source | Genbank ID | Status | Size | Genes | CDS | G+C |
| --- | --- | --- | --- | --- | --- | --- | --- | --- |
| *C. bowmanii* | DSM 14206 | Microbial mat | GCA_018861315.1 | Draft | 4.885 | 4637 | 4531 | 31.2 |
| *C. estertheticum* | CEST001 | Lamb | GCA_013093435.1 | Draft | 4.807 | 4678 | 4563 | 30.9 |
| *C. estertheticum* | CF001 | Bovine feces | GCA_019537155.1 | Draft | 4.825 | 4689 | 4586 | 30.9 |
| *C. estertheticum* | CF002 | Bovine feces | GCA_018861295.1 | Draft | 5.331 | 5179 | 5071 | 30.6 |
| *C. estertheticum* | CF003 | Bovine feces | GCA_018861435.1 | Draft | 5.322 | 5079 | 4972 | 30.9 |
| *C. estertheticum* | CF004 | Bovine feces | GCA_019661245.1 | Draft | 4.991 | 4838 | 4722 | 30.9 |
| *C. estertheticum* | CF005 | Bovine feces | GCA_019661205.1 | Draft | 5.112 | 5011 | 4892 | 30.5 |
| *C. estertheticum* | CF006 | Bovine feces | GCA_018861375.1 | Draft | 5.101 | 4962 | 4845 | 30.9 |
| *C. estertheticum* | CF007 | Bovine feces | GCA_019661225.1 | Draft | 4.993 | 4796 | 4692 | 30.8 |
| *C. estertheticum* | CF008 | Bovine feces | GCA_019537135.1 | Draft | 4.525 | 4377 | 4270 | 30.7 |
| *C. estertheticum* | CF009 | Bovine feces | GCA_018861325.1 | Draft | 5.227 | 5061 | 4954 | 30.6 |
| *C. estertheticum* | CF010 | Bovine feces | GCA_018861855.1 | Draft | 5.167 | 5074 | 4970 | 30.5 |
| *C. estertheticum* | CF013 | Bovine feces | GCA_018861795.1 | Draft | 5.192 | 5047 | 4932 | 30.6 |
| *C. estertheticum* | CF015 | Bovine feces | GCA_020443505.1 | Draft | 5.501 | 5387 | 5160 | 30.7 |
| *C. estertheticum* | CF016 | Bovine feces | GCA_020443575.1 | Draft | 5.409 | 5215 | 4981 | 30.6 |
| *C. estertheticum* | CM018 | Lamb | GCA_018861665.1 | Draft | 4.896 | 4663 | 4552 | 30.6 |
| *C. estertheticum* | CM020* | Lamb | GCA_018861305.1 | Draft | 4.898 | 4665 | 4554 | 30.6 |
| *C. estertheticum* | CM032* | Beef | GCA_020443525.1 | Draft | 4.811 | 4726 | 4606 | 30.6 |
| *C. estertheticum* | CM033* | Horse meat | GCA_020443465.1 | Draft | 4.814 | 4698 | 4495 | 30.6 |
| *C. estertheticum* | CM034 | Horse meat | GCA_020443485.1 | Draft | 4.805 | 4694 | 4505 | 30.6 |
| *C. estertheticum* | CM035 | Lamb | GCA_020443435.1 | Draft | 5.073 | 4939 | 4746 | 30.5 |
| *C. estertheticum* | DSM 14864 | Beef | GCA_008933175.1 | Draft | 4.994 | 4811 | 4658 | 30.5 |
| *C. estertheticum* | DSM 8809 | Meat | GCA_001877035.1 | Complete | 4.785 | 4550 | 4409 | 30.9 |
| *C. estertheticum* | MA19* | Pork | GCA_009295575.1 | Draft | 4.965 | 4693 | 4623 | 30.5 |
| *C. estertheticum* | MA41 | Pork | GCA_009295545.1 | Draft | 4.971 | 4965 | 4618 | 30.5 |
| *C. estertheticum* | DSM 17811 | Microbial mat | GCA_018861905.1 | Draft | 5.188 | 5029 | 4914 | 30.8 |
| *C. frigoris* | DSM 14204 | Microbial mat | GCA_018861865.1 | Draft | 4.121 | 3915 | 3794 | 30.5 |
| *C. lacusfryxellense* | DSM 14205 | Microbial mat | GCA_018861735.1 | Draft | 5.405 | 5168 | 5059 | 31.1 |
| *C. psychrophilum* | DSM 14207 | Permafrost | GCA_018861705.1 | Draft | 4.116 | 3897 | 3772 | 29.9 |
| *C. algoriphilum* | DSM 16153 | Permafrost | To be submitted | Draft | 4.520 | 4623 | 4524 | 30.7 |
| *C. tagluense* | CM008 | Lamb | GCA_019537185.1 | Draft | 5.325 | 5130 | 5027 | 31.1 |
| *C. tagluense* | CM019* | Lamb | GCA_020443605.1 | Draft | 5.310 | 5079 | 4889 | 30.9 |
| *C. tagluense* | CM021* | Lamb | GCA_020443795.1 | Draft | 5.311 | 5043 | 4853 | 30.9 |
| *C. tagluense* | CM022 | Lamb | GCA_020443665.1 | Draft | 5.310 | 5037 | 4856 | 30.9 |
| *C. tagluense* | CM023* | Beef | GCA_020443545.1 | Draft | 5.312 | 5045 | 4851 | 30.9 |
| *C. tagluense* | CM024 | Beef | GCA_018861655.1 | Draft | 5.207 | 4971 | 4860 | 31.0 |
| *C. tagluense* | CM025* | Lamb | GCA_020443745.1 | Draft | 5.310 | 5090 | 4987 | 30.9 |
| *C. tagluense* | CM031* | Lamb | GCA_020443725.1 | Draft | 5.311 | 5045 | 4933 | 30.9 |
| *C. tagluense* | CF014* | Bovine feces | GCA_020443645.1 | Draft | 5.310 | 5083 | 4894 | 30.9 |
| *C. tagluense* | CS002 | Equipment | GCA_020443745.1 | Draft | 5.415 | 5144 | 4968 | 30.9 |
| *C. tagluense* | DSM 17763 | Permafrost | GCA_003865095.1 | Draft | 5.195 | 5298 | 5181 | 30.9 |
| *C. tagluense* | FP1 | Lamb | GCA_011065975.1 | Draft | 5.379 | 5339 | 5148 | 31.1 |
| *C. tagluense* | FP2 | Venison | GCA_011065955.1 | Draft | 5.549 | 5454 | 5271 | 30.9 |
| *Clostridium* spp. | CM027 | Lamb | GCA_019537145.1 | Draft | 3.769 | 3583 | 3478 | 31.7 |
| *Clostridium* spp. | CM028 | Lamb | GCA_019537175.1 | Draft | 3.662 | 3439 | 3337 | 31.6 |
| *Clostridium* spp. | CF011 | Bovine feces | GCA_018861595.1 | Draft | 3.872 | 3684 | 3578 | 31.5 |
| *Clostridium* spp. | CF012 | Bovine feces | GCA_018861755.1 | Draft | 5.445 | 5369 | 5241 | 31.7 |
| *Clostridium* spp. | CS001 | Equipment | GCA_020443565.1 | Draft | 4.139 | 3943 | 3776 | 31.6 |
| *Clostridium* spp. | FP3 | Lamb | GCA_011065935.1 | Draft | 5.555 | 5374 | 5208 | 31.5 |
| *Clostridium* spp. | FP4 | Lamb | GCA_011065905.1 | Draft | 4.088 | 4032 | 3881 | 31.3 |

*The genomes of the strains were excluded from the previous genome mining study (Wambui et al., 2022) to avoid an overestimation of bacteriocin biosynthetic gene clusters. Readers are directed to Wambui et al., (2022) for further details.

**Reference**

Wambui, J., Stevens, M. J. A., Sieber, S., Cernela, N., Perreten, V., & Stephan, R. (2022). Targeted genome mining reveals the psychrophilic *Clostridium estertheticum* complex as a potential source for novel bacteriocins, including cesin A and estercticin A. *Frontiers in Microbiology*, *12*. https://doi.org/10.3389/fmicb.2021.801467

**Supplementary Table 1B**. Description of species and strain names used in the current study in accordance with those in the NCBI database

| NCBI database | | Current study | |
| --- | --- | --- | --- |
| Species | Strain | Species | Strain |
| *Clostridium* spp. | CM027 | Genomospecies2 | CM027 |
| *Clostridium* spp. | CM028 | Genomospecies2 | CM028 |
| *Clostridium* spp. | CF011 | Genomospecies2 | CF011 |
| *Clostridium* spp. | CF012 | Genomospecies3 | CF012 |
| *Clostridium* spp. | CS001 | Genomospecies4 | CS001 |
| *Clostridium* spp. | FP3 | Genomospecies1 | FP3 |
| *Clostridium* spp. | FP4 | Genomospecies2 | FP4 |

**Supplementary Table 2**: List of genes used for mapping the acetone-butyrate-ethanol (ABE) fermentation pathway and hydrogenases in *C. estertheticum* and *C. tagluense*. The EC numbers correspond with KEGG database while the gene function correspond with the annotation in RAST webserver. The NCBI accession numbers are provided for each encoded protein.

| Gene | EC number | Function | NCBI accession number |
| --- | --- | --- | --- |
| *ldh* | EC 1.1.1.27 | L-lactate dehydrogenase (EC 1.1.1.27) | WP_071611441.1 |
| *alss* | EC 2.2.1.6 | Acetolactate synthase large subunit (EC 2.2.1.6) | WP_071612557.1 |
| *alss* | EC 2.2.1.6 | Acetolactate synthase small subunit (EC 2.2.1.6) | WP_071612558.1 |
| *pfor* | EC 1.2.7.1 | Pyruvate-flavodoxin oxidoreductase | WP_071612569.1 |
| *fnor-nfn* |  | NADH-dependent reduced ferredoxin:NADP+ oxidoreductase subunit A/B | WP_071611155.1/WP_071611156.1 |
| *pta* | EC 2.3.1.8 | Phosphate acetyltransferase (EC 2.3.1.8) | WP_071613249.1 |
| *ack* | EC 2.7.2.1 | Acetate kinase (EC 2.7.2.1) | WP_071613248.1 |
| *adh* | EC 1.1.1.1 | Acetaldehyde dehydrogenase (EC 1.2.1.10) / Alcohol dehydrogenase (EC 1.1.1.1) | WP_071612172.1 |
| *thl* | EC 2.3.1.9 | Acetyl-CoA acetyltransferase (EC 2.3.1.9) | WP_071613581.1 |
| *hbd* | EC 1.1.1.157 | 3-hydroxybutyryl-CoA dehydrogenase (EC 1.1.1.157) | WP_071611153.1 |
| *crt* | EC 4.2.1.55 | 3-hydroxybutyryl-CoA dehydratase (EC 4.2.1.55) | WP_071611149.1 |
| *bcd/etf* |  | Electron bifurcating butyryl-CoA dehydrogenase (NAD+, ferredoxin) | WP_071611150.1 |
| *bcd/etf* |  | Electron bifurcating butyryl-CoA dehydrogenase, electron transfer flavoprotein beta | WP_071611151.1 |
| *bcd/etf* |  | Electron bifurcating butyryl-CoA dehydrogenase, electron transfer flavoprotein alpha | WP_071611152.1 |
| *ptb* | EC 2.3.1.19 | acetobutylicum phosphotransbutyrylase | WP_071610962.1 |
| *buk* | EC 2.7.2.7 | Butyrate kinase (EC 2.7.2.7) | WP_125002980.1 |
| *ctfA/B* | EC 2.8.3.9 | Butyrate--acetoacetate CoA-transferase subunit A (EC 2.8.3.9) | WP_071611623.1 |
| *ctfA/B* | EC 2.8.3.9 | Butyrate--acetoacetate CoA-transferase subunit B (EC 2.8.3.9) | WP_071611624.1 |
| *bdh* |  | NADH-dependent butanol dehydrogenase | WP_071612325.1 |
| *pfl* | EC 2.3.1.54 | Pyruvate formate-lyase (EC 2.3.1.54) | WP_071614328.1 |
| *hydA* |  | [FeFe] hydrogenase (EC 1.12.7.2) | WP_071614690.1 |
| *hydE* |  | [FeFe]-hydrogenase maturation protein HydE | WP_071612789.1 |
| *hydF* |  | [FeFe]-hydrogenase maturation protein HydF | WP_071611297.1 |
| *hydG* |  | [FeFe]-hydrogenase maturation protein HydG | WP_071611758.1 |
| *hyaA* | EC 1.12.1.4 | Bifurcating [FeFe] hydrogenase, alpha subunit (EC 1.12.1.4) | WP_071612029.1 |
| *hyaB* | EC 1.12.1.4 | Bifurcating [FeFe] hydrogenase, beta subunit (EC 1.12.1.4) | WP_071612028.1 |
| *hyaC* | EC 1.12.1.4 | Bifurcating [FeFe] hydrogenase, gamma subunit (EC 1.12.1.4) | WP_071612027.1 |
| *hycA* |  | Hydrogenase-4 component B | WP_071611123.1 |
| *hycB* |  | Hydrogenase-4 component C | WP_071611124.1 |
| *hycC* |  | hydrogenase, membrane subunit 2-like protein | WP_071611125.1 |
| *hycD* |  | Hydrogenase-4 component F | WP_071611126.1 |
| *hycE* |  | Hydrogenase-4 component G | WP_071611127.1 |
| *hycF* |  | Formate hydrogenlyase subunit 7-like protein | WP_071611128.1 |
| *fdh* | EC 1.2.1.2 | Formate dehydrogenase-O, major subunit (EC 1.2.1.2) | WP_164948303.1 |
| *hypA* |  | [NiFe] hydrogenase nickel incorporation protein HypA | WP_164947888.1 |
| *hypB* |  | [NiFe] hydrogenase nickel incorporation-associated protein HypB | WP_216276216.1 |
| *hypC* |  | [NiFe] hydrogenase metallocenter assembly protein HypC | WP_216276219.1 |
| *hypD* |  | [NiFe] hydrogenase metallocenter assembly protein HypD | WP_164948973.1 |
| *hypE* |  | [NiFe] hydrogenase metallocenter assembly protein HypE | WP_220321717.1 |
| *hypF* |  | [NiFe] hydrogenase metallocenter assembly protein HypF | WP_164948971.1 |
| *hypG* |  | [Ni/Fe] hydrogenase, group 1, small subunit | WP_220321716.1 |
| *hypH* |  | [Ni/Fe] hydrogenase, group 1, large subunit | WP_220321715.1 |
| *hypI* |  | Hydrogenase maturation protease delta subunit, HyaD-like | WP_220321714.1 |
| *hybA* | EC 1.12.7.2 | Periplasmic [Fe] hydrogenase large subunit (EC 1.12.7.2) | WP_220321470.1 |
| *hybB* | EC 1.12.7.2 | Periplasmic [FeFe] hydrogenase large subunit (EC 1.12.7.2) | WP_220321472.1 |
| *hybC* |  | HoxF-like protein | WP_164949696.1 |
| *hybD* |  | HoxE-like protein | WP_164949577.1 |

**Supplementary Table 3**. Variation of *Clostridium estertheticum* complex pangenome based on BLAST cut-off

|  | BLAST cut-off (%) | | | | |
| --- | --- | --- | --- | --- | --- |
|  | 60 | 70 | 80 | 90 | 95 |
| Core (99% ≤ strains ≤ 100%) | 1071 | 1004 | 748 | 185 | 32 |
| Soft core (95%≤ strains ≤ 100%) | 1367 | 1228 | 942 | 262 | 55 |
| Shell (15% ≤ strains < 95%) | 6421 | 6611 | 7296 | 8783 | 8971 |
| Cloud (0% ≤ strains < 15%) | 21849 | 23767 | 28741 | 43255 | 57749 |


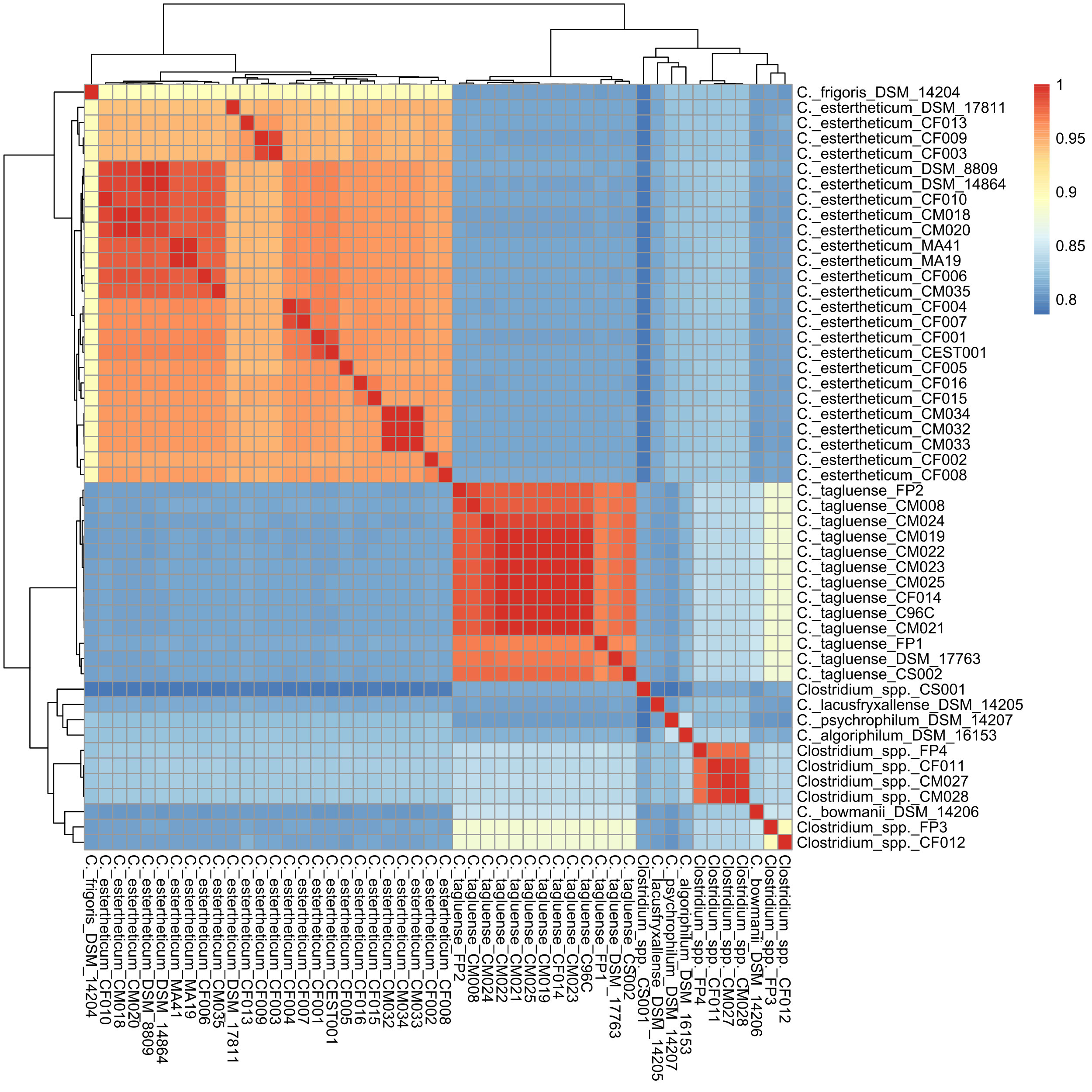


**Supplementary Figure 1**. ﻿Heat map of average nucleotide identity (ANIb) analysis of 50 sequenced *Clostridium estertheticum* complex strains.


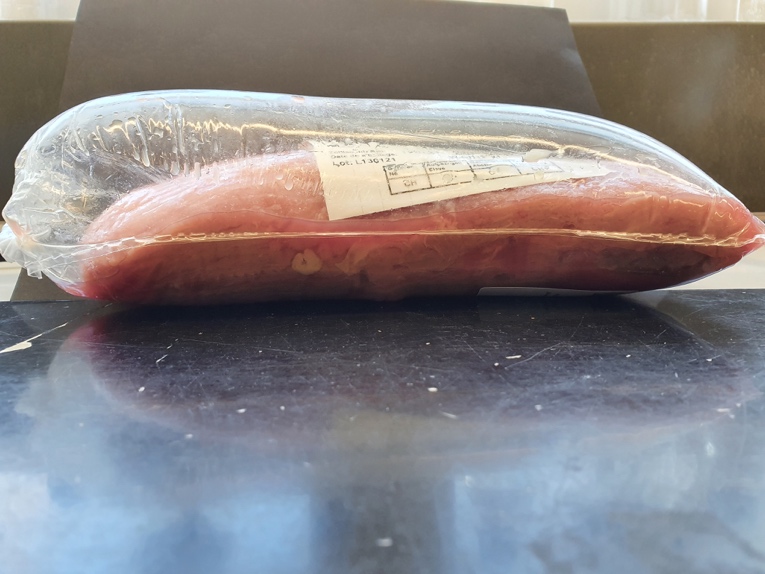


**Supplementary Figure 2**. Blown pack spoilage case of beef that was caused by *Clostridium estertheticum* CM032


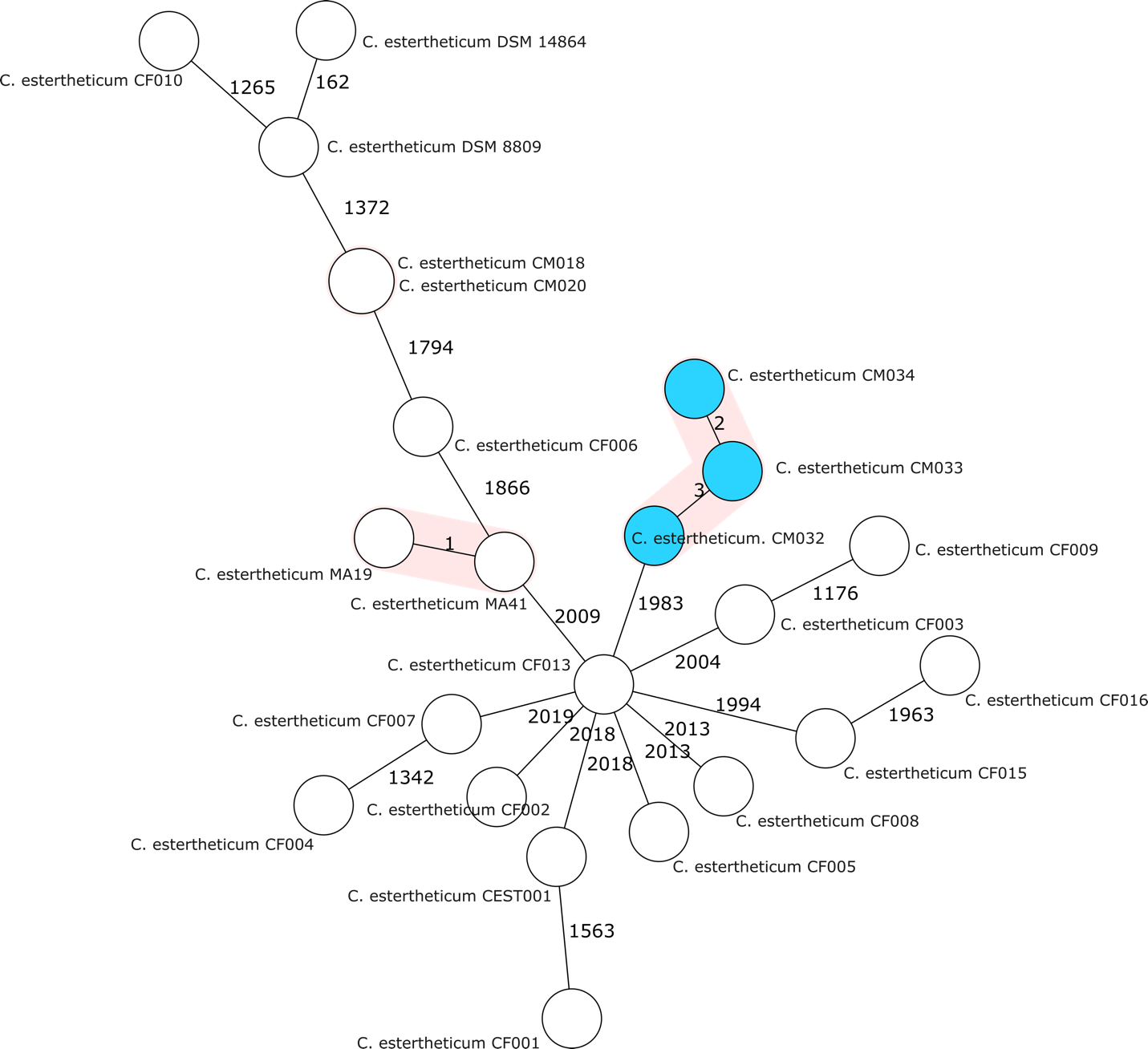


**Supplementary Figure 3**. cgMLST of *Clostridium estertheticum* species. The three strains (highlighted in blue) causing blown pack spoilage in beef (CM032) and horse meat (CM033 and CM034) clustered together and had a SNP difference of ≤3 indicating they are clonal despite differences in isolation source.


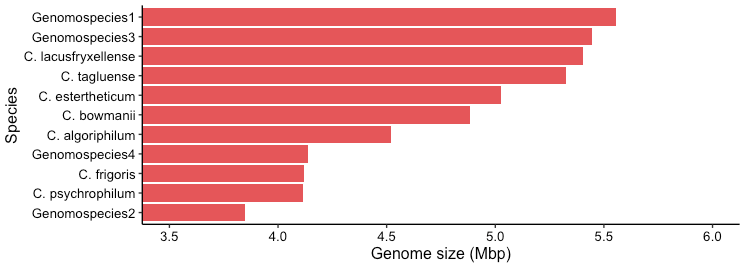


**Supplementary Figure 4**. Average genome size of *Clostridium estertheticum* complex species.


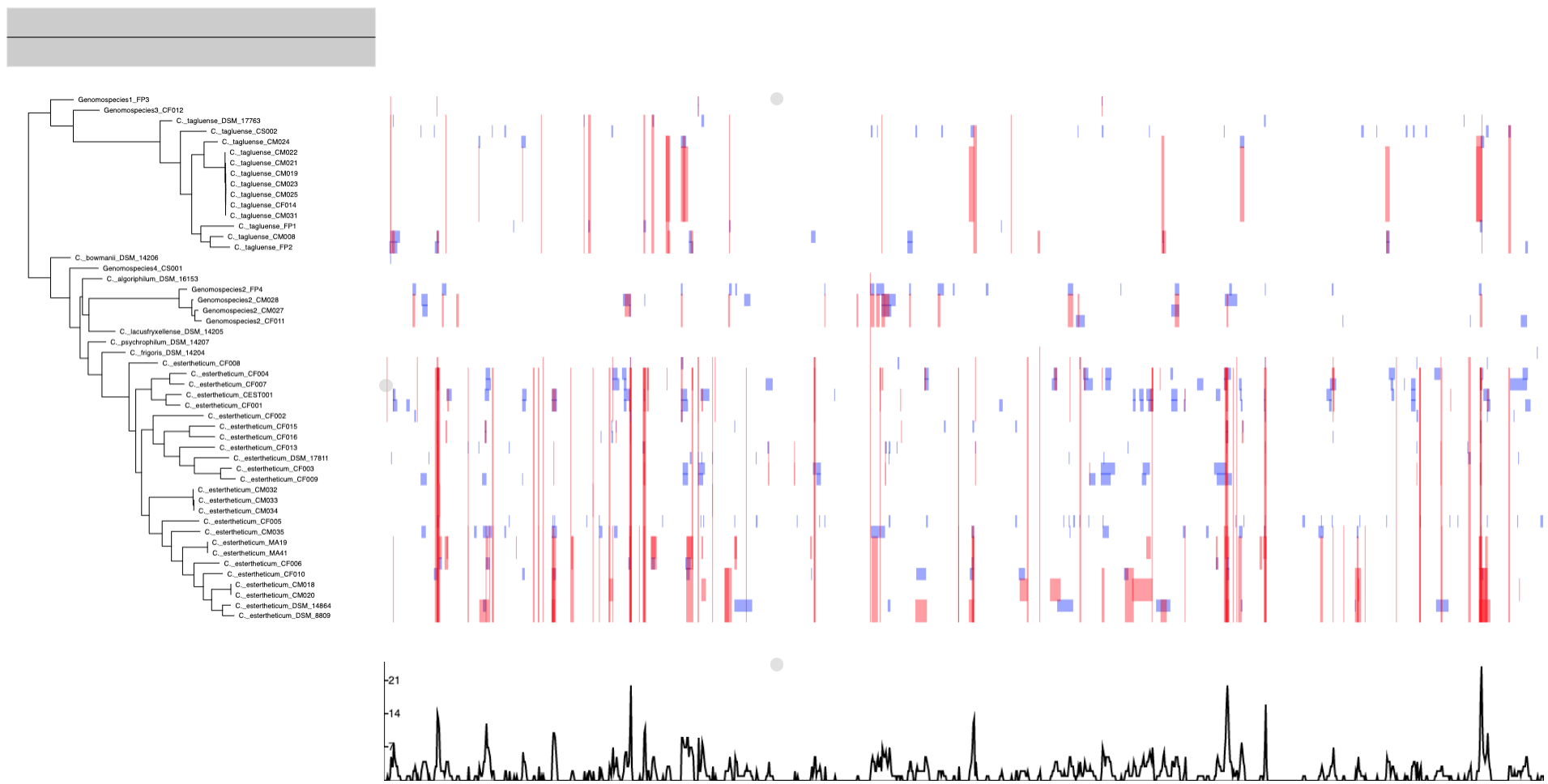


A

B

C

**Supplementary Figure 5.** Recombination hotspots in *Clostridium estertheticum* complex. (A) The core-genome-based phylogenetic tree of CEC. (B) The detected recombination hotspots: red blocks represent ancestral recombination while the blue blocks represent specific recombination to one isolate. (C) The bottom part of the figure corresponds to a graph measuring SNP density.


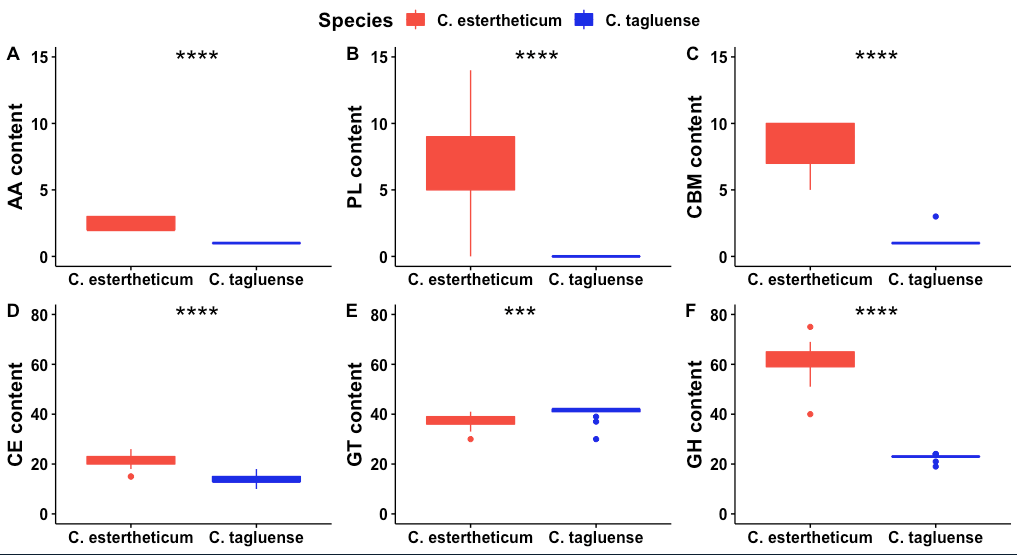


**Supplementary Figure 6**. Variable distribution of carbohydrate metabolizing genes between *Clostridium estertheticum* and *C. tagluense*


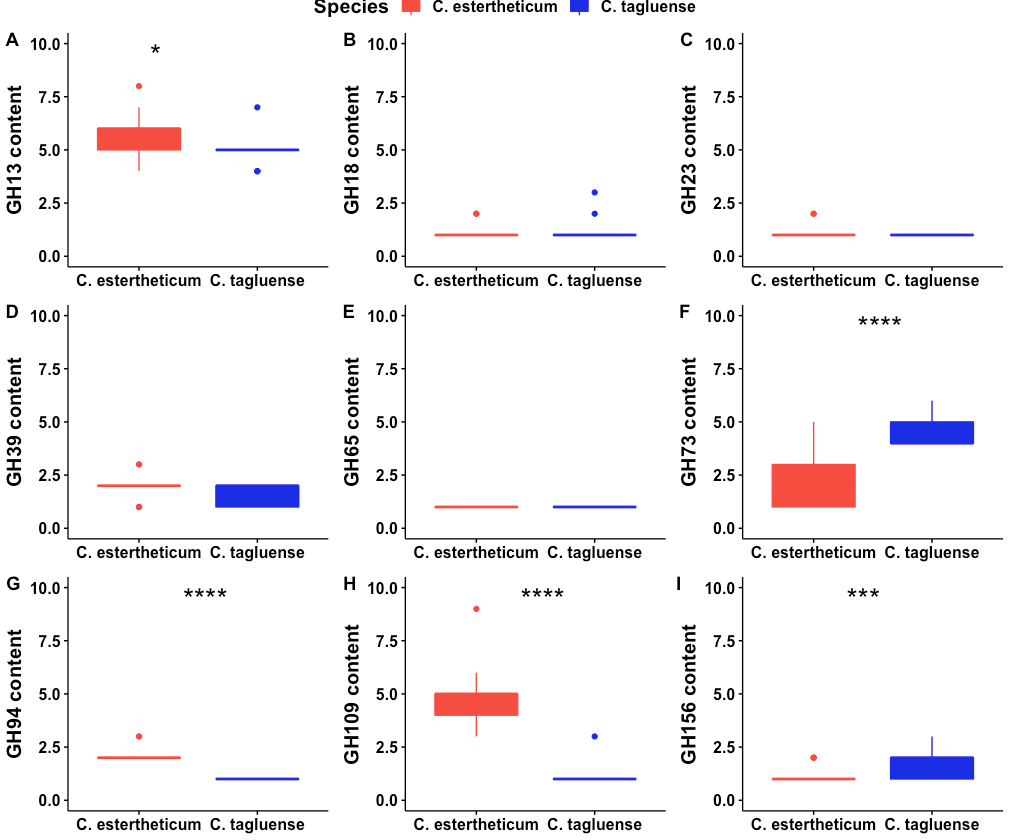


**Supplementary Figure 7**. Variable distribution of Glycoside hydrolases that are present in all genomes of *Clostridium estertheticum* and *C. tagluense*


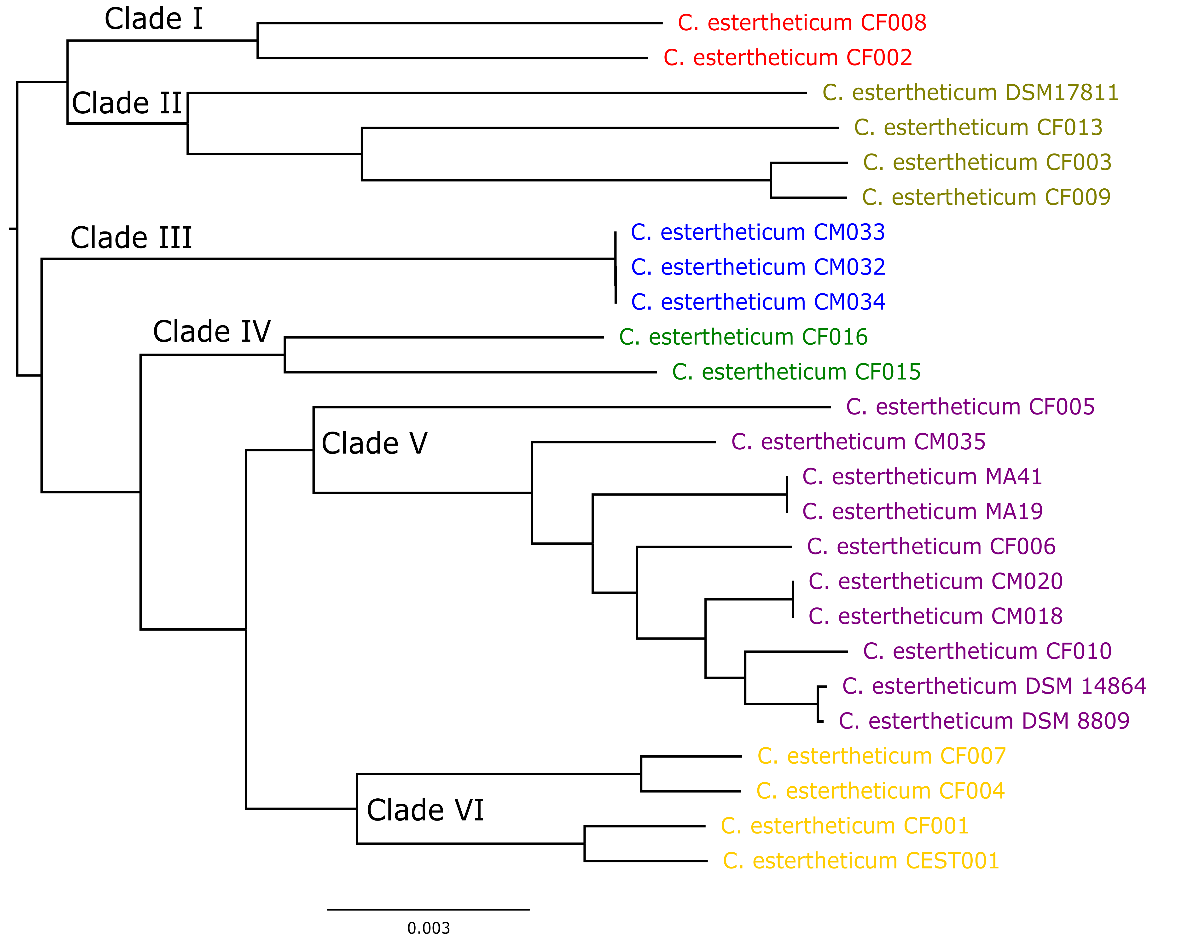


**Supplementary Figure 8**. Core-genome based phylogeny of *Clostridium estertheticum*. The species could be divided into six putative clades highlighted in different colors. The phylogenetic tree was made from aligned genomes using RAxML
